# Visual prompt engineering for multimodal and irregularly sampled medical data

**DOI:** 10.1038/s43856-026-01817-x

**Published:** 2026-09-01

**Authors:** Malte Tölle, Mohamad Scharaf, Samantha Fischer, Christoph Reich, Silav Zeid, Christoph Dieterich, Benjamin Meder, Norbert Frey, Philipp Wild, Sandy Engelhardt

**Affiliations:** 1 Department of Cardiology, Angiology and Pneumology, Heidelberg University Hospital, Heidelberg, Germany; 2 Informatics for Life Institute, Heidelberg, Germany; 3 DZHK (German Centre for Cardiovascular Research), Partner Site Heidelberg/Mannheim, Heidelberg, Germany; 4 Preventive Cardiology and Preventive Medicine, Department of Cardiology, University Medical Center of the Johannes Gutenberg University Mainz, Mainz, Germany; 5 Clinical Epidemiology and Systems Medicine, Center for Thrombosis and Hemostasis, University Medical Center Mainz, Johannes Gutenberg University Mainz, Mainz, Germany; 6 DZHK (German Centre for Cardiovascular Research), Partner Site Rhine-Main, Mainz, Germany; 7 Systems Medicine, Institute of Molecular Biology (IMB), Mainz, Germany

**Keywords:** Medical imaging, Diagnosis

## Abstract

**Background:**

A patient undergoes multiple examinations in each hospital stay, where each provides different facets of the health status. These assessments include temporal data with varying sampling rates, discrete single-point measurements, therapeutic interventions such as medication administration, and images. While physicians are able to process and integrate diverse modalities intuitively, neural networks need specific modeling for each modality complicating the training procedure.

**Methods:**

We demonstrate that this complexity can be significantly reduced by visualizing all information as images along with unstructured text and subsequently training a conventional vision-text transformer. Our approach, **Vi**sion **T**ransformer for **i**rregular sampled **M**ulti-modal **M**easurements (**ViTiMM**), simplifies data preprocessing and modeling by unifying clinical measurements, medications, X-ray images, and electrocardiography scans into a single visual representation.

**Results:**

ViTiMM outperforms current state-of-the-art methods in predicting in-hospital mortality, phenotyping, and decompensation on two datasets, the MIMIC-IV and COVID Data for Shared Learning (CDSL) dataset.

**Conclusions:**

We hope our work inspires advancements in multi-modal medical AI by reducing the training complexity to (visual) prompt engineering, thus lowering entry barriers and enabling no-code solutions for training. The source code is publicly available at https://github.com/Cardio-AI/ViTiMM.

## Plain language summary

During a hospital stay, doctors gather many types of information about a patient, including blood tests, output from heart monitors, details of medications, and X-ray images. Whilst doctors are trained and able to combine all of these different data types when making decisions, this is difficult for computers, as each type of information usually requires its own specialized approach for processing. In this study, we simplified this problem by converting all patient data into images and text, which can then be processed by a single artificial intelligence model called ViTiMM. We tested our approach on two large patient datasets and found that it outperforms existing methods in predicting important outcomes such as whether a patient’s condition will worsen. Our work makes it easier to build medical AI systems, potentially lowering technical barriers and accelerating future clinical applications.

## Abstract (web summary)

Tolle et al. propose ViTiMM, a method that visualizes all clinical data modalities as images to simplify multi-modal medical AI training using a conventional vision-text transformer. It outperforms some other approaches for mortality prediction, phenotyping, and decompensation across two intensive care datasets.

## Introduction

**Fig. 1:**
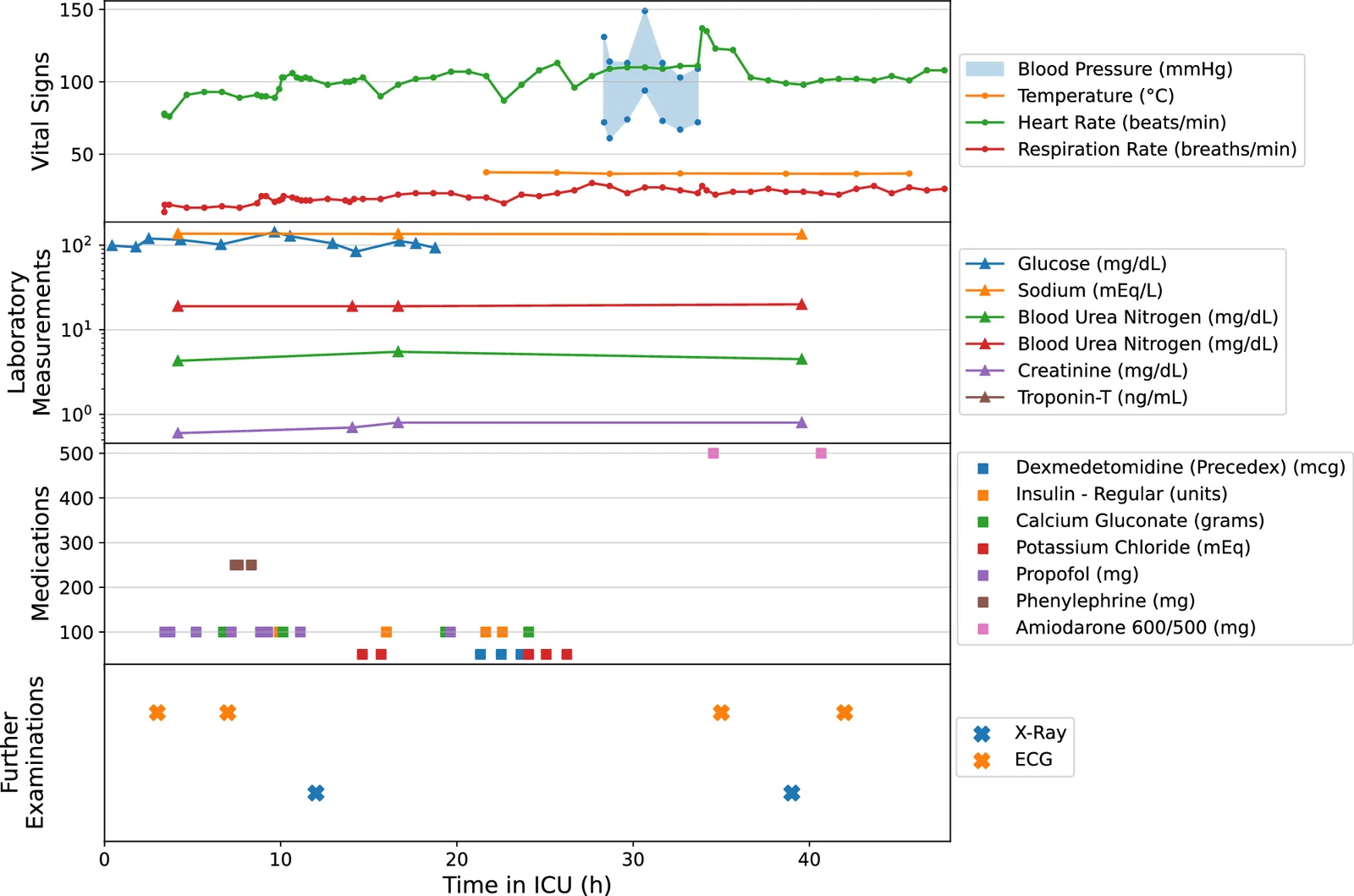
Example patient journey in the first 48 h in the intensive care unit for hadm_id 28016813 from the MIMIC-IV dataset^22^. The patient examinations include, among others, vital signs (e.g., blood pressure), laboratory measurements (e.g., glucose), medications (e.g., insulin), and additional tests such as X-ray images and electrocardiography scans. The measurements are recorded at irregular time intervals, which differ between patients, adding further complexity to the modeling process. Additionally, medications can be administered in various doses, which must also be considered.

During a hospital stay, a patient typically undergoes multiple examinations, each offering distinct insights into their health status. While physicians have learned to intuitively extract the different information and assemble them to an overall picture, neural networks need specific modeling of the different modalities and their interactions. Nevertheless, once these challenges are addressed, multi-modal models have demonstrated promising performance^1–8^. However, a significant challenge persists: How to integrate multi-modal data that is captured at irregularly sampled time intervals (Fig. 1).

Among the examinations conducted for a patient are laboratory measurements conducted at irregular time intervals to monitor vital organ functions, detect abnormalities, and guide treatment decisions. These tests provide critical information on parameters such as electrolyte balance, blood gases, and metabolic status. When a patient’s values deviate from the ideal range, a variety of medications, often prepared in different solutions, are administered based on the patient’s specific needs. Other often but irregularly performed examinations include X-ray to monitor conditions such as heart failure or bone status and electrocardiography (ECG) scans to assess the electrical activity of the heart. Physicians are then tasked with integrating all the partial information from different, irregular time points into a comprehensive picture to evaluate whether the patient requires additional care.

Multi-modal deep learning in medicine holds the potential to reduce the workload of physicians in such cases but faces a significant challenge: the integration of multiple modalities into a single model due to differing modeling requirements^1,9^. In conventional approaches, each modality requires a distinct model architecture or embedding, which must then be fused to generate outputs^4^. For imaging modalities, features can be extracted using techniques such as convolutional neural networks and subsequently concatenated with features from another imaging modality^10,11^ or with tabular patient metadata^12,13^. When incorporating additional modalities, such as ECG, a separate model architecture must be utilized^14,15^. In recent years, transformer models^16^ have simplified the modeling process, as the attention mechanism provides a general framework for handling diverse modalities^14,17^. Despite this, different modalities still require unique embedding mechanisms^18,19^. Another promising technique is contrastive learning, which enables models to learn similar representations across modalities^20,21^. This approach has demonstrated strong performance in tasks such as radiology report generation when trained on paired X-ray and text samples from the MIMIC-IV dataset^2,22^. However, contrastive learning requires substantial amounts of data to effectively capture underlying data representations.

Our primary contribution is a substantial reduction of the modeling complexity for multiple irregularly sampled modalities by transforming each modality into an image representation. Humans are then tasked with visualizing the different modalities in an informative manner, effectively engaging in a form of “visual prompt engineering”. For example, laboratory measurements can be represented as line graphs over time to convey trends and patterns^23^. Our approach, **Vi**sion **T**ransformer for **i**rregular sampled **M**ulti-modal **M**easurements (**ViTiMM**), unifies the data processing pipeline, significantly reducing modeling complexity. This approach not only mimics the way humans interpret diverse data streams but also demonstrates significant improvements across a range of tasks.

Among the multiple modalities, especially irregularly sampled time series data (e.g., laboratory measurements in medicine) require special attention. In a multivariate setting, most methods assume regularly and evenly spaced observational intervals, but these may not necessarily overlap. Thus, the observations are commonly converted to continuous-time observations with fixed intervals or explicitly modeling the relationship between sensors^24–26^. While these approaches have shown good performances, the effort can significantly be reduced by displaying the time series as line graphs and subsequently train a conventional vision transformer (ViT) for classification^23^. This not only renders the need for additional modeling obsolete but also shows superior performance. Our approach builds upon the seminal work and extends the setting to the multi-modal case. We show that the information from multiple modalities can be easily combined without the need for explicit modeling by using conventional vision-text transformer^21,27^. We show that we can integrate a multitude of modalities, more precisely clinical parameters such as e.g., laboratory measurements, data from bedside monitors, and the given medications, ECG scans, and X-ray images by representing each modality as an image and feeding the additional information (e.g., patient metadata) as text to the model. By displaying each modality as an image (i.e., line graphs), we harmonize the feature extraction pipeline across inputs and acquire multi-modality at no additional cost. Further, optimally visualizing modalities as images can be interpreted as visual prompt engineering, which demands significantly less effort and coding expertise compared to designing specialized architectures for specific use cases. This approach lowers barriers to entry, facilitating no- or low-code solutions.

In the literature, the Medical Transformer (MeTra)^28^, medical multi-modal fusion (MedFuse)^29^, Missing-modality enabled multi-modal fusion architecture (MMFusion)^30^ have been proposed for working with clinical time-series data as well as chest X-ray images from the MIMIC-IV dataset. MeTra deals with the irregular intervals by taking a day-wise average for each variable and feeding the resulting vector per patient to a linear layer before merging with the extracted features from the X-ray image. MedFuse discretizes and interpolates the EHR values for harmonized presentation to the model. They propose to use a long-short term memory (LSTM) network to fuse the extracted features from both modalities, which is particularly useful for the partial instead of paired presence of input modalities per patient. MMFusion employs a transformer based bi-modal fusion framework together with multivariate loss functions. The fusion architecture combines features extracted from domain-specific pre-trained models (DenseNet121^31^, PubMedBERT^32^), which are subsequently processed through a transformer-based fusion module.

Compared to the approaches in the literature, the contribution of our work is five-fold:

- We are the first to train on more than two modalities from the MIMIC-IV^22^ and the Covid Data for Shared Learning (CDSL)^33^ dataset, including laboratory measurements, medications, electrocardiograms, and X-ray images. Previous multi-modal approaches have been limited by the complexity of modeling more than two modalities.
- We significantly reduce this modeling complexity by representing each modality as an image. This approach unifies the data extraction pipeline and facilitates low- to no-code solutions, with the training process primarily influenced by a form of visual prompt engineering.
- We outperform three state-of-the-art methods, MMFusion^30^, MeTra^28^, and MedFuse^29^, across datasets (MIMIC-IV and CDSL) and tasks such as in-hospital mortality, and decompensation prediction as well as phenotyping. Our results demonstrate that incorporating more modalities improves model performance.
- We enhance the interpretability of our model by visualizing attention maps, which highlight the input regions most influential in the model’s predictions.
- We open source our code, model weights, and example scripts, providing accessible tools to apply our method to other datasets with minimal coding effort.

## Methods

This manuscript’s study and results adhere to all pertinent ethical guidelines and uphold ethical standards in both research conduct and manuscript preparation, in accordance with all relevant laws and regulations concerning human subject treatment. All models were trained on publicly available datasets described below.

### Data and benchmarks

Few open medical datasets provide extensive multi-modal information. We evaluate on two such datasets: the Medical Information Mart for Intensive Care (MIMIC-IV)^22,34^ and the COVID Data for Shared Learning (CDSL) dataset. Both offer detailed clinical records with linked imaging modalities such as X-ray^35^ and ECG^36^. This study was performed in accordance with the Declaration of Helsinki. Both datasets used in this study are publicly available, de-identified, and accessed via PhysioNet. No additional IRB or ethics-committee approval was sought or required for the present study, because it is a secondary analysis of existing, publicly available, de-identified datasets and involves no contact with patients, no collection of new data, and no access to identifiable information; under these conditions the work does not constitute human-subjects research requiring further review.

#### MIMIC-IV

The collection of patient information and creation of the research resource was reviewed by the Institutional Review Board at the Beth Israel Deaconess Medical Center, who granted a waiver of informed consent and approved the data sharing initiative. PhysioNet The IRB protocol number is 2001-P-001699 CrossmarkarXiv. The dataset was also approved by the IRB of the Massachusetts Institute of Technology (No. 0403000206). All data were de-identified in compliance with the HIPAA Safe Harbor provision.

The most frequently used dataset with matched time-resolved samples on a patient level across multiple modalities is the Medical Information Mart for Intensive Care (MIMIC)^22^. MIMIC-IV is a freely accessible dataset that contains extensive clinical information from de-identified patients admitted to the emergency department or an intensive care unit at Beth Israel Deaconess Medical Center (BIDMC) in Boston, Massachusetts, between 2008 and 2019. It consists of two main modules. The hospital-wide EHR module includes patient and admission information, medication administrations, billed diagnoses, microbiological data, laboratory measurements and hospital performance-related information. The ICU-specific module comprises detailed records to ICU stays, including intravenous and fluid administrations, patient excretions, ventilation data, and clinical interventions. In total, the dataset contains 299,712 unique patient identifiers, 431,231 hospitalizations and 73,181 intensive care unit (ICU) stays. All data is publicly available via Physionet^34^.

For subsets of the patients contained in the MIMIC-IV dataset further modalities are published, more precisely chest X-ray (CXR) images (MIMIC-CXR)^35,37^ as well as ECG scans (MIMIC-IV-ECG)^36^. The MIMIC-CXR dataset includes 377,110 CXR images (frontal and lateral views) corresponding to 227,835 radiological examinations collected between 2011 and 2016. The radiographs are in DICOM format and sourced from the hospital’s Picture Archiving and Communication System (PACS). A free-text radiology report is assigned to each examination. MIMIC-IV-ECG provides electrocardiograms from 161,352 different patients and includes 800,035 diagnostic ECGs recorded between 2008 and 2019. While 55% can be linked to a hospitalization record and 25% to a visit to the emergency department, for 20% no link to the MIMIC-IV dataset can be established.

To provide rigorous evaluation to other methods, benchmark tasks need to be defined, of which three exist that were also used in other works^28,29,38^.

- *In-hospital mortality***:** This binary classification task aims to predict in-hospital mortality based on the first 48 h of ICU stay. Patients with ICU stays shorter than 48 h are excluded.
- *Phenotype classification*: In this multi-label classification task a set of 25 chronic, mixed, and acute care conditions are assigned to a patient in an ICU stay (e.g., acute myocardial infarction or shock).
- *Decompensation*: This binary classification task aims to predict whether a patient will experience clinical decompensation within the subsequent 24-h period of their ICU stay.

In all tasks, the instance is paired with the last CXR image and ECG scan during the first 48 h of the ICU stay. Table 1 shows the resulting dataset splits with their respective quantities. The age of the patients ranges from 18 to 91 years with an average of 64 ± 16 years. Approximately 55% of patients were male. To enable to comparison to MeTra we only use samples that have paired EHR and CXR data. Still, not all of these patients have an ECG scan, which highlights the possible extensibility of our method to unpaired samples.

**Table 1:** Summary of matched MIMIC-IV, -CXR, and -ECG datasets and the corresponding splits

Variable	All	Train set	Validation set	Test set
Patients	6125	4396	472	1257
Unique Stay IDs	6798	4885	540	1373
CXR Images	6798 (100%)	4885 (100%)	540 (100%)	1373 (100%)
ECG Scans	3840 (56%)	2753 (56%)	301 (56%)	786 (57%)
Clinical Parameters	6798 (100%)	4885 (100%)	540 (100%)	1373 (100%)
In-hospital mortality	1002 (16%)	717 (16%)	76 (16%)	209 (17%)
Decompensation	365 (5%)	260 (5%)	24 (4%)	81 (6%)

The phenotyping labels are present for all patients, their individual prevalence can be found in Supplementary Table 7.

#### CDSL

The COVID Data for Shared Learning (CDSL) dataset received approval for release from the HM Hospitales Clinical Research Ethics Committee, with internal authorization code auth_opendata_cdsl3_170624. PhysioNet: The data were de-identified prior to release. As this study exclusively uses publicly available, de-identified datasets accessed through signed data use agreements, and no additional patient data were collected, no separate ethics approval was required. The authors completed the required training in human research (CITI) and signed the PhysioNet Credentialed Health Data Use Agreement.

The CDSL dataset^33^ is a multimodal database containing de-identified medical data from 4479 patients hospitalized with confirmed or suspected COVID-19 at HM Hospitales in Spain between December 2019 and February 2021. Unlike MIMIC-IV, CDSL was specifically designed for COVID-19 research and includes comprehensive clinical information alongside 4608 chest X-rays in JPG format. The dataset comprises structured EHR data including demographics, diagnoses, vital signs (224,146 records), medications (115,649 records), and laboratory results (786,984 tests), all linked by unique patient identifiers. However, CDSL lacks ECG data and pre-defined benchmark tasks, necessitating adaptation of evaluation protocols from established datasets. For consistency with MIMIC-IV evaluation, we implemented the in-hospital mortality prediction task using the first five days of hospitalization data. The dataset is publicly available via PhysioNet following ethical approval. The age of the patients ranges from 0 to 106 years with an average of 67 ± 17 years. Approximately 59% of patients were male.

### Clinical rationale for multi-modal fusion

The clinical data modalities used in this work, namely laboratory values, electrocardiograms, chest X-rays, medications, and patient metadata, capture fundamentally different physical quantities: biochemical concentrations, cardiac electrical potentials, thoracic pixel intensities, treatment decisions, and demographic context, respectively.

For *in-hospital mortality prediction*, laboratory values such as lactate, creatinine, and blood gases reflect acute organ dysfunction and metabolic derangement^39,40^, which are among the strongest individual short-term mortality predictors. ECG data reveal cardiac arrhythmias, ischemia, or conduction abnormalities that signal cardiovascular instability entirely absent from laboratory panels^41^. Chest X-rays identify structural findings such as pulmonary edema, pneumothorax, or pleural effusions^42^, reflecting cardiopulmonary compromise not directly measurable through blood chemistry or electrical signals. Medication data encodes the clinical team’s implicit assessment of severity (e.g., vasopressor use indicating hemodynamic instability)^43^, providing a proxy for acuity that none of the other modalities capture directly.

For *phenotyping*, different diagnoses manifest in fundamentally different modalities^44,45^: atrial fibrillation is primarily visible in the ECG, pneumonia is identifiable on chest X-ray, metabolic disorders such as acute kidney injury are reflected in laboratory values, and certain conditions are best inferred from treatment patterns (e.g., anticoagulant prescription suggesting thromboembolic disease). No single modality covers the full ICD codespace, so combining them necessarily expands the set of conditions the model can detect.

For *decompensation prediction*, clinical deterioration typically involves a cascade across organ systems with temporally staggered, modality-specific warning signs^46,47^: subtle laboratory trends (rising lactate, dropping platelets), worsening cardiac function visible in ECG changes, fluid overload becoming apparent on chest X-ray, and medication escalation (e.g., initiation of vasopressors) often preceding measurable physiological deterioration. A model with access to all modalities can therefore detect deterioration earlier and more reliably than one limited to a single data source.

### Model input transformations

**Fig. 2:**
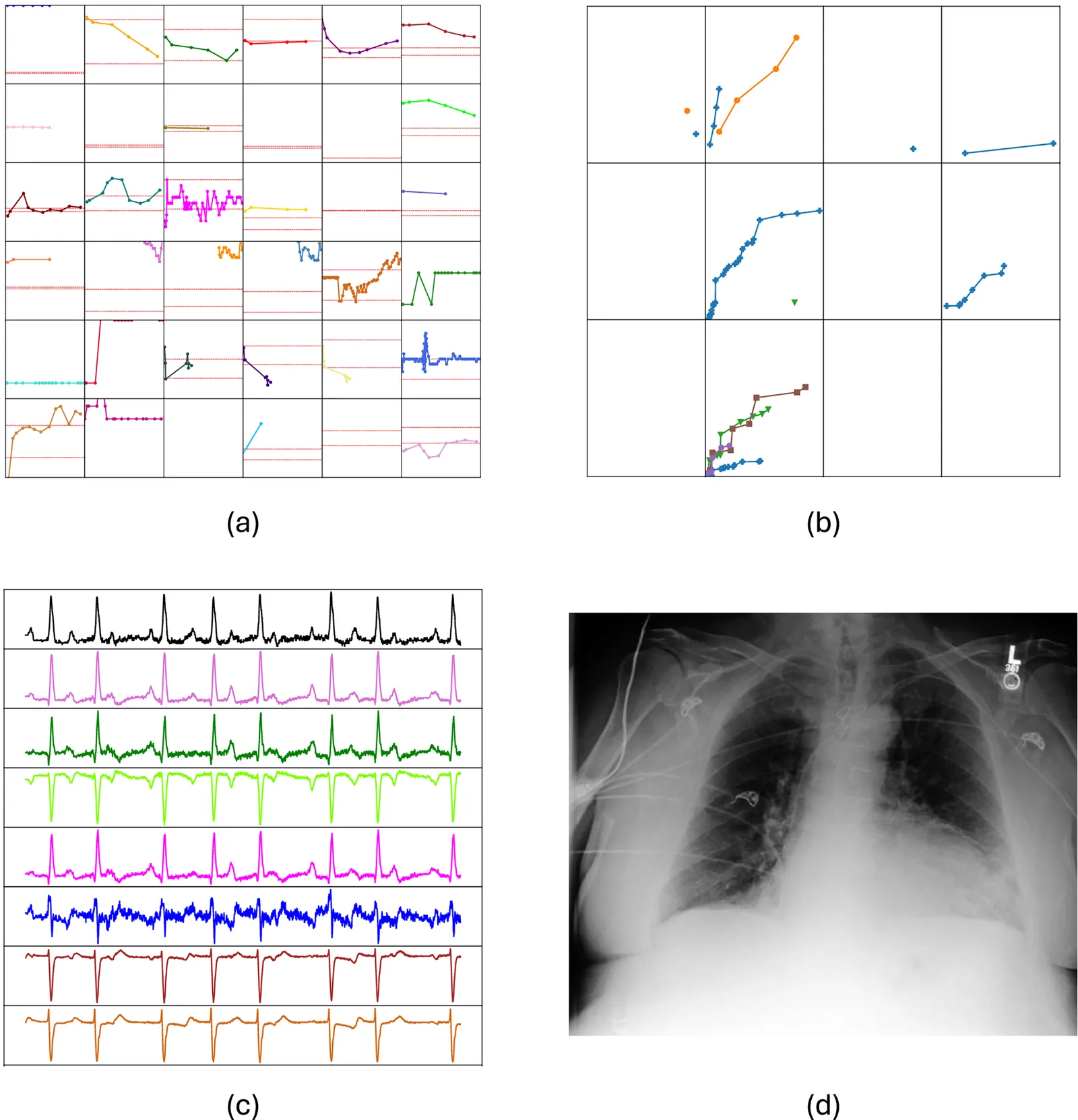
The four modalities from the MIMIC-IV dataset. We tested various formats (e.g., removing “*” markings, preprocessing ECGs), and found the visualized format performed best. **a** Clinical measurements with the *x*-axis representing 48 h. **b** Medications as cumulative dosage of the different drugs taken. **c** 12-lead ECG with minimal preprocessing, from which 8 are shown. **d** CXR image. The field names for clinical measurements can be found in Supplementary Fig. 1 and for medications in Supplementary Fig. 2. The 12 leads of the ECG are in standard chronological order^36^.

To enable training on time series data with vision transformers a transformation into images more precisely line graphs is necessary. All three time series modalities need different preprocessing to be afterwards processed in a similar way. Only X-ray scans need no further preprocessing as they are already in image format (Fig. 2d).

#### Irregularly sampled time series line graphs

To visualize the course of temporal data points most often line graphs are used, where each observation is marked by its time (*x*-axis) and value (*y*-axis). These observations are connected with straight lines in chronological order, which accounts for interpolating values in between. This approach is agnostic to the type of time series under consideration and generalizes as we show for different modalities. Essentially, this is a form of visual prompt engineering similar to language models, where users refine and optimize natural language prompts to improve model performance^23^. To distinguish measurements from interpolation we use a “*" symbol to indicate observations. We plot each variable in a separate plot with a distinct color for better differentiation as was found beneficial for model training (see Fig. 2a)^23^. We omit tick labels as well as other graphical components as an observation’s position already signals its relative time and magnitude.

Following the notation from ref. ^23^, let ${{{\mathcal{D}}}}=\left\{({{{{\mathcal{C}}}}}_{i},{{{{\mathcal{M}}}}}_{i},{{{{\mathcal{X}}}}}_{i},{{{{\mathcal{E}}}}}_{i},{{{{\mathcal{T}}}}}_{i},{y}_{i})| i=1,\ldots ,N\right\}$ denote our multimodal dataset containing *N* samples, where ${{{\mathcal{C}}}}$ represents clinical measurements, ${{{\mathcal{M}}}}$ medications, ${{{\mathcal{X}}}}$ X-ray images, ${{{\mathcal{E}}}}$ ECG data, and ${{{\mathcal{T}}}}$ text data. For each irregularly sampled time series *S*_*i*_, observations for variable *d* are given by sequences of tuples $[({t}_{1}^{d},{v}_{1}^{d}),({t}_{2}^{d},{v}_{2}^{d}),\ldots ,({t}_{{n}_{d}}^{d},{v}_{{n}_{d}}^{d})]$, where *n*_*d*_ is the number of observations. Our framework comprises: (1) a transformation function converting time series *S*_*i*_ into image *x*_*i*_, and (2) a vision transformer classifier predicting label ${\hat{y}}_{i}$. Given a set of time series line graphs *G*_*i*_ = *g*_1_, *g*_2_, . . . , *g*_*D*_ for a time series *S*_*i*_, we arrange them in a single image xi using a pre-defined grid layout. We adopt a square grid by default. Specifically, we arrange the D time series line graphs in a grid of size *l* × *l* if *l* × (*l* − 1) < *D*≤*l* × *l*, and a grid of size *l* × (*l* + 1) if *l* × *l* < *D*≤*l* × (*l* + 1).

#### Clinical measurements

We identified 36 different clinical variables for MIMIC and 25 for CDSL in consultation with expert physicians (Supplementary Tables 3 and 4). Before plotting, each variable is standardized to zero mean and unit variance with (*x* − *μ*)/*σ*. Subsequently, each variable was displayed in its own quadrant with a distinct color. If no measurements were present for a specific patient the quadrant is left blank. Supplementary Tables 3 and 4 show the missing ratios for all parameters. Each variable is consistently visualized in the same quadrant and color for all patients. An example is shown in Fig. 2a, the corresponding variable names can be found in Supplementary Figs. 1 and 3. For providing further guidance to the model for each variable the normal lower and upper range are visualized as red lines in each quadrant.

#### Medications

Compared to clinical measurements, visualizing medications required more effort. Different prescriptions could be given in different solutions, which were encoded in different drug names. So, we first grouped the medications into ten categories, such as e.g., beta-blockers or antiarrhythmics and assigned different substances into the categories (Supplementary Tables 3, 5, and 6. Each category received one quadrant in the final block, whereas each substance within a category was a separate line graph with a consistent color across all patients (Fig. 2b and Supplementary Figs. 2 and 4. For each substance the cumulative dosage is shown, i.e., each line is consistently increasing over time. Similar to clinical measurements to remove outliers we clip the cumulative values at the 95th quartile. Afterwards all cumulative amounts are normalized between 0 and 1 with the maximum amount across all patients. Due to the high number of different administered drugs (e.g., different solution combinations) each medication category exhibits high missing ratios.

#### Electrocardiography scans

We sampled the waveforms at 500 Hz and performed standardization^14^. We extracted 5s from the signal due to limited input resolution. Since ECGs are regularly sampled a visualization in line graphs is straightforward (Fig. 2c). To preserve important details each of the twelve leads is plotted in a new row. As for clinical measurements and medications we also resort to assigning a specific color to each lead for better differentiation.

#### Patient metadata

To enable learning on categorical patient metadata we feed important information as text to our language model^48^. These include the patient’s sex, age, and ethnicity as well as insurance status. Further, we feed the findings and impressions from each CXR report, the machine measurements i.e., diagnoses from the ECG such as e.g., myocardial infarction or bundle branch blocks, and the diagnoses encoded in the international classification of diseases (ICD) codes. Last, we also feed the prescribed medications as string without the administered dosages as we found this improves predictive performance.

### Architecture with vision transformers

The line graphs involve both local (the temporal course of a single variable) and global (the correlation among several variables) contexts. Given enough data vision transformers have been shown to excel at maintaining spatial information and stronger capabilities to capture both, local and global contexts^17,49,50^. The conventional vision transformer (ViT) splits the image into fixed size patches that are subsequently linearly projected and extended with position embeddings^17^. The resulting vectors together with a dedicated classification token are fed into a multi-head self attention modules. Global context is modeled with attention between all pairs of patches, which can become computationally expensive. Swin (shifted window) transformer on the other hand trades of local and global inter-unit interactions by formulating a hierarchical representation^23^. Small-sized patches in earlier layers and gradually merged with neighboring ones deeper in the network. Self-attention is computed within each non-overlapping window, which allows for capturing local intra-variable interactions and temporal dynamics of a single line graph i.e., one variable^23^. By shifting the window for creating patches in the subsequent layer and merging the attention connection between different windows the capture of global context i.e., interactions between different variables is enabled. Formally, consecutive Swin Transformer blocks alternate between window-based multi-head self-attention (W-MSA) and shifted window multi-head self-attention (SW-MSA), each followed by an MLP block with layer normalization (LN) and residual connections^23,49^.

**Fig. 3:**
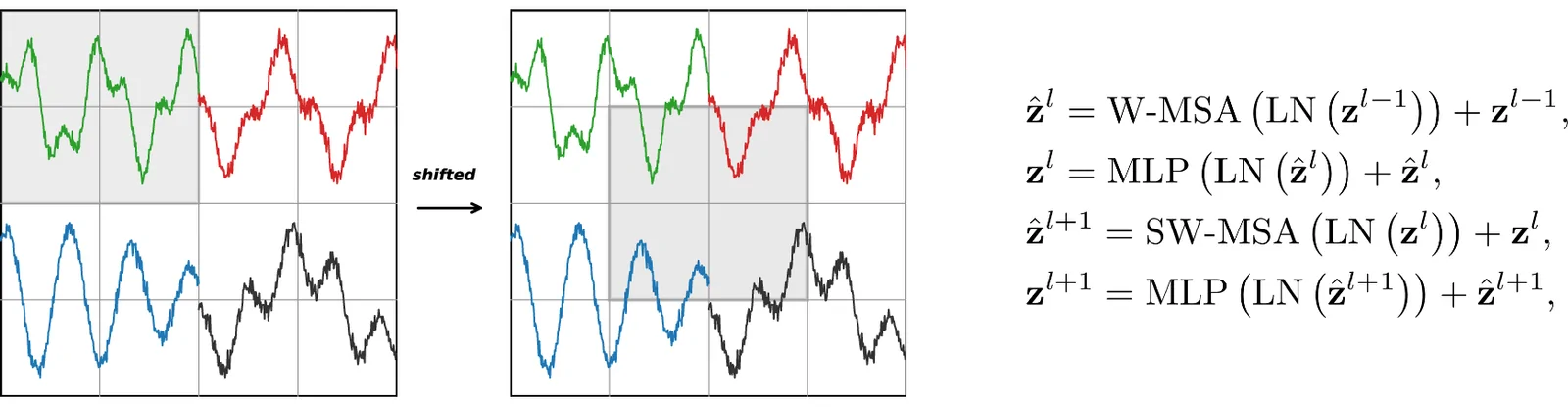
Shifted window mechanism in the Swin Transformer applied to stacked line graphs of different variables. Self-attention is computed within non-overlapping windows (gray region). In the left panel, the window resides within a single pair of line graphs, capturing local temporal dynamics. After shifting (right panel), the window spans across line graphs of different variables, enabling the modeling of global cross-variable dependencies^23,49^. ${\hat{{{{\bf{z}}}}}}^{l}$ and **z**^*l*^ denote the output features of the (S)W-MSA module and the MLP module for block *l*, respectively.

**Fig. 4:**
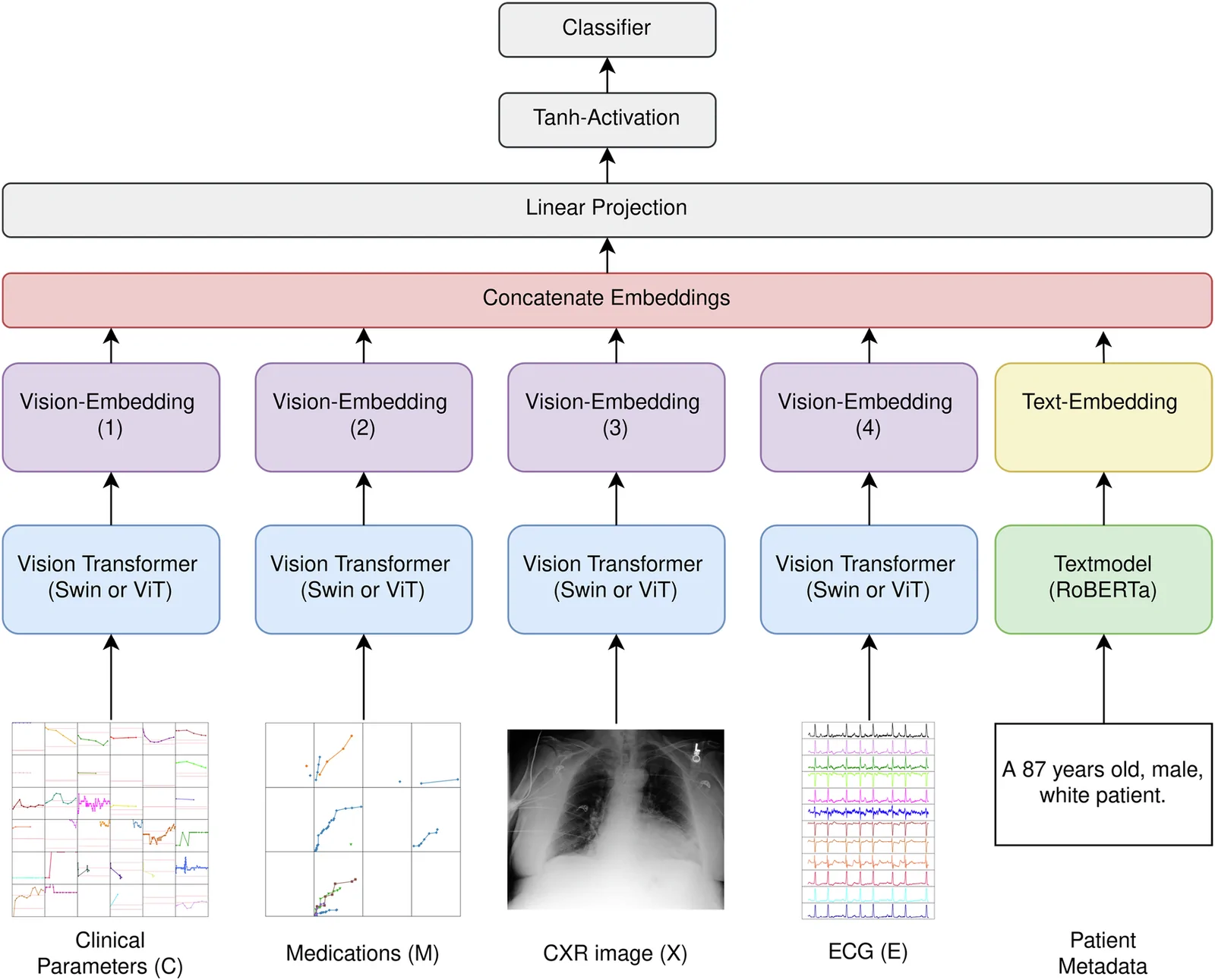
Multi-modal medical vision transformer with arbitrary modalities as images. Each modality is represented as an image i.e., clinical measurements, administered medications, and electrocardiography scans are displayed as line graphs. No changes must be made to CXR images. Each image can be fed into a vision transformer (conventional ViT^17^ or SWIN^49^). Additionally, patient metadata is fed as text into a RoBERTA model^48^. As embeddings are added after feature extraction, arbitrary modalities can be added or subtracted.

When a window is contained within a single line graph, W-MSA captures local temporal dynamics of that variable, while after shifting, SW-MSA connects patches across different line graphs, enabling the modeling of global cross-variable correlations (see Fig. 3) ^23^. At the final stage, patch representations are flattened and passed through a linear classification head to obtain per-modality predictions, following Li et al.^23^. We rely on the pretrained positional embeddings without additional local or global position encodings, as Li et al. found no consistent improvement from such additions, suggesting that the original ImageNet-pretrained embeddings already capture sufficient spatial information^23^. For each modality, we use a separate Swin transformer^49^ pretrained on ImageNet^51^. The patient metadata is fed into a RoBERTa text model^48^ similar to Li et al.^23^. The extracted features are concatenated before being linearly projected and fed into a final classification layer (Fig. 4). While early fusion approaches are also possible, we deliberately chose late fusion as this allows for seamless addition and subtraction of modalities by only changing the number of input features in the linear projection layer. Early fusion on the other hand would require architectural changes within the feature extractors. Unless otherwise specified we use an image size of 384 × 384 with channel-wise normalization taken from ImageNet^51^. For the text model, we chose a Byte-Pair Encoding (BPE) tokenizer with a context length of 512 tokens, consistent with the input requirements of the RoBERTa architecture.

### Baseline comparisons

To enable rigorous evaluation of our method, we compare against two baseline methods from the literature that attempted to solve the same tasks, in-hospital mortality prediction and phenotyping on the MIMIC-IV dataset^22^, name MedFuse^29^, MeTra^28^, and missing-modality enabled multi-modal fusion (MMFusion)^30^. All methods work on (paired) CXR images and electronic health records (EHR) i.e., clinical measurements. In MeTra, the CXR images are processed by a conventional ViT that converts the images into latent representations (tokens). For each of the clinical measurements a mean over 48 hours is taken, if a parameter is missing it is filled with the overall mean. The resulting vector is projected with a linear layer. Both representations are concatenated and fed into four multi-head self attention blocks^16^, before being processed by a classification layer. MedFuse processes the CXR images with a ResNet34 encoder^52^. The time series data from the clinical measurements is fed to a two-layer long short-term memory (LSTM) network^53^. To also enable learning on unpaired data, one or both representation is fed sequentially into a one-layer LSTM. MMFusion employs a transformer-based bi-modal fusion framework^30^. The method processes features from pre-trained domain-specific encoders (DenseNet121^31^ for medical imaging and PubMedBERT^32^ for clinical text) through transformer blocks that can adaptively weight available modalities when one or more data sources are missing during inference.

## Results

From the MIMIC-IV dataset^22^, three standard benchmarks are extracted^38^. All task are based on the data from the first 48 hours of a patient’s ICU stay. In the first task, *in-hospital mortality* shall be predicted, in the second a collection of 25 diagnosed conditions must be classified (*phenotyping*), and in the third it shall be assessed whether the patient *decompensates* within the next 24 h. From the CDSL dataset^33^, we also extract the *in-hospital mortality* task in the same manner. We compare our model, the Vision Transformer for irregular sampled Multi-modal Measurements (ViTiMM), to two approaches from the literature, namely Multimodal Medical Transformer MMFusion^30^, (MeTra)^28^, and MedFuse^29^. However, all methods are only implemented for clinical measurements together with CXR images. No method exists so far that extends the training task to further modalities such as the administered medications as well as a recorded ECG scan during the ICU stay. To create maximal compatibility all approaches are only trained on data for which paired clinical measurements and CXR images are available. The results are summarized in Tables 2 and 3. The dataset sizes are shown in Tables 1 and 4 in the “Methods” section.

**Table 2:** Results of ViTiMM for the task *In-hospital Mortality* on the MIMIC-IV^22^ and CDSL^33^ dataset compared to MMFusion^30^, MeTra^28^, and MedFuse^29^

		MIMIC: In-hospital Mortality			CDSL: In-hospital Mortality		
Method	Modalities	AUROC	AUPRC	Bal.Acc.	AUROC	AUPRC	Bal.Acc.
MeTra^28^	C	0.791	0.441	0.609	0.506	0.228	0.500
	X	0.810	0.471	0.544	0.667	0.307	0.624
	*L*∣*X*	0.859	0.595	0.707	0.724	0.352	0.648
MedFuse^29^	C	0.812	0.448	0.571	0.641	0.265	0.500
	X	0.662	0.264	0.500	0.638	0.280	0.500
	*L*∣*X*	0.805	0.431	0.631	0.690	0.233	0.500
MMFusion^30^	C	0.537	0.187	0.494	0.910	0.578	0.842
	X	0.720	0.298	0.613	0.914	0.524	0.842
	*L*∣*X*	0.731	0.320	0.648	0.905	0.569	0.846
ViTiMM (Ours)	C	0.837	0.512	0.743	0.986	0.900	0.948
	X	0.826	0.494	0.758	0.958	0.689	0.914
	M	0.741	0.346	0.680	0.942	0.662	0.908
	E	0.704	0.297	0.636	-	-	-
	*C*∣*X*	**0.875**	**0.615**	**0.776**	**0.987**	**0.933**	**0.937**
	*C*∣*M*∣*X*(∣*E*)	**0.922**	**0.764**	**0.847**	**0.987**	**0.934**	**0.958**

We compare the three methods for uni-modal training for clinical measurements (C) and X-ray (X) as well as a combination of the two similar to their original publication. Extension of both methods to further modalities requires explicit modeling, which must not be done in ViTiMM. Thus, by only plotting the other modalities our method can straightforward expand to arbitrary modalities. The best method per task and metric is highlighted in bold. The corresponding significance tests (pairwise t-test) can be found in Supplementary Tables 9 and 12. An ablation study for the other modality combinations can be found in Supplementary Table 8.

*C* clinical measurements, *X* CXR images, *M* medications, *E* electrocardiography.

**Table 3:** Results of ViTiMM for the task *Decompensation* and *Phenotyping* from the MIMIC-IV dataset^22^ compared to MMFusion^30^, MeTra^28^, and MedFuse^29^

		MIMIC: Decompensation			MIMIC: Phenotyping		
Method	Modalities	AUROC	AUPRC	Bal.Acc.	AUROC	AUPRC	Bal.Acc.
MeTra^28^	C	0.703	0.167	0.498	0.691	0.400	0.574
	X	0.668	0.160	0.500	0.667	0.387	0.564
	*L*∣*X*	0.712	0.168	0.492	0.712	0.431	0.583
MedFuse^29^	C	0.748	0.262	0.539	0.705	0.417	0.569
	X	0.651	0.115	0.500	0.640	0.349	0.538
	*L*∣*X*	0.765	0.343	0.560	0.733	0.448	0.600
MMFusion^30^	C	0.528	0.114	0.500	0.615	0.341	0.549
	X	0.688	0.158	0.515	0.603	0.333	0.526
	*L*∣*X*	0.715	0.194	0.621	0.644	0.372	0.564
ViTiMM (Ours)	C	0.670	0.186	0.500	0.766	0.506	0.618
	X	0.781	0.246	0.500	0.730	0.460	0.589
	M	0.681	0.181	0.500	0.710	0.430	0.577
	E	0.634	0.163	0.500	0.681	0.427	0.573
	*C*∣*X*	**0.797**	**0.329**	**0.663**	**0.778**	**0.530**	**0.636**
	*C*∣*M*∣*X*∣*E*	0.793	0.277	0.540	**0.784**	**0.549**	**0.659**

The best method per task and metric is highlighted in bold. The results per phenotype can be found in Supplementary Table 7. The corresponding significance tests (pairwise t-test) can be found in Supplementary Tables 10 and 11.

*C* clinical measurements, *X* CXR images, *M* medications, *E* electrocardiography.

**Table 4:** Summary of COVID Data for Shared Learning (CDSL) dataset and the corresponding splits

Variable	All	Train set	Validation set	Test set
Patients	4406	3207	317	882
CXR images	1311 (30%)	994 (31%)	82 (26%)	235 (27%)
Clinical parameters	4387 (99%)	3193 (99%)	316 (99%)	878 (99%)
In-hospital mortality	538 (12%)	403 (13%)	41 (13%)	94 (11%)

All experiments were conducted on a single NVIDIA RTX 3090 GPU with 24 GB VRAM, where the Swin transformer with resolution 384 × 384 demands 464.692 GFLOPs per forward pass. Inference times demonstrate real-time applicability, it achieves 0.074 s for all modalities. Environmental impact was quantified using Carbon Tracker ^54^. For the CDSL dataset, single modality training required approximately 6 minutes 30 seconds, consuming 0.076 kWh energy and generating 28.82 g CO_2_ emissions. Complete multimodal training extended to 10 min 30 s with 0.129 kWh energy consumption and 49.05 g CO_2_ emissions. On the larger MIMIC dataset, single modality training took 9 min 45 s, consuming 0.128 kWh and producing 48.77 g CO_2_. Full multimodal training required approximately 27 min with 0.224 kWh energy consumption and 85.46 g CO_2_ emissions.

### Uni-modal training

First, all methods are evaluated for single modality training. MeTra and MedFuse are designed to work with only two modalities. However, our method easily accommodates training on administered medications and ECG scans by converting their time-series data into visual plots, while utilizing the exact same training procedure as for clinical measurements and CXR images.

#### MIMIC In-hospital mortality

For clinical measurements (C), our method achieves the highest area under receiver operating curve (AUROC) of 0.837 (MeTra: 0.791, MedFuse: 0.812) and balanced accuracy (Bal.Acc. = (sensitivity + specificity)/2) of 0.743 (MeTra: 0.609, MedFuse: 0.571). For area under the precision recall curve (AUPRC) MeTra achieves a value of 0.441, MedFuse 0.448, and ours 0.512. Classifying CXR images (X) mainly comes down to choosing the better feature extractor. Here, we can see the superiority of the Swin transformer^49^ in our method (AUROC = 0.826) compared to the conventional ViT^17^ in MeTra (0.810) and a ResNet34^52^ in MedFuse (0.662). Training on administered medications (M) and ECG scans (E) yields lower classification accuracy. For medications our method achieves a AUROC of 0.741 (AUPRC = 0.346, Bal.Acc. = 0.680), for ECG the AUROC is 0.704 (AUPRC = 0.297, Bal.Acc. = 0.636).

#### MIMIC phenotyping

Phenotyping, a multi-label multi-class binary classification task for present diagnoses, is a more challenging task than predicting in-hospital mortality. However, our method, ViTiMM, outperforms MeTra and MedFuse in the single modality setting for both, clinical measurements (C) and CXR images (X) (see Table 3). By training on C we achieve an AUROC of 0.766 (MeTra: 0.691, MedFuse: 0.705), with training on X the scores are slightly lower: ViTiMM: 0.730, MeTra: 0.667, MedFuse: 0.644. Again, this is mostly determined by the employed architecture.

#### MIMIC decompensation

Similar to in-hospital mortality, decompensation represents a binary classification task. In contrast to the other prediction tasks, chest X-ray images appear to contain more discriminative information for decompensation detection, as evidenced by consistently superior performance when training on imaging data (*X*) compared to clinical measurements (*C*). For instance, MMFusion achieves an AUROC of 0.688 on X-ray data versus 0.528 on clinical measurements, while ViTiMM demonstrates even greater improvement with 0.781 versus 0.670, respectively. This pattern suggests that radiological features provide more predictive signals for identifying early signs of clinical deterioration than traditional vital signs and laboratory values alone.

#### CDSL In-hospital mortality

**Fig. 5:**
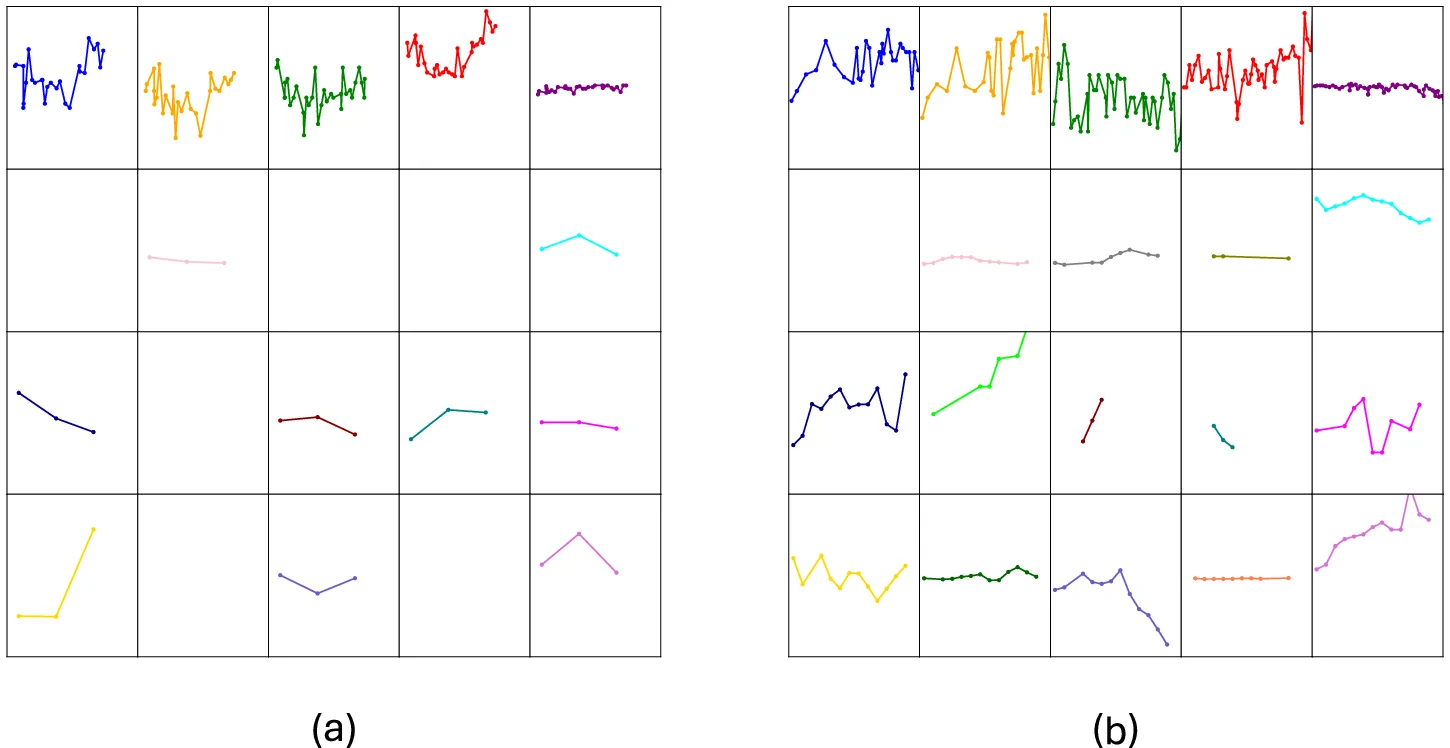
Example plots of clinical parameters for two patients in the CDSL dataset^33^. While the patient in (**a**) survived the hospital stay, the patient in (**b**) did not. When comparing the two plots, it becomes evident that the vital signs (first row) show considerably greater variability for the patient who died during the hospital stay. This pattern of increased physiological instability is observed across most patients in the dataset. The variable names for all clinical measurements can be found in Supplementary Fig. 1, an example for a medications plot together with the variable names is presented in Supplementary Fig. 2.

Despite the extended temporal resolution compared to MIMIC-III (five days vs. 48 h), both MMFusion and ViTiMM demonstrate exceptional performance on in-hospital mortality prediction using the CDSL dataset, achieving AUROCs of 0.914 and 0.986, respectively. This superior performance can be attributed to the pronounced fluctuations observed in patients’ vital signs, particularly among those who experienced in-hospital mortality (Fig. 5). Our analysis reveals that patients with fatal outcomes exhibit significantly greater variability in their physiological parameters throughout their hospital stay. Notably, this pattern creates a highly discriminative signal that these models can effectively leverage highlighting that our method captures the global context of the signal. However, when presented with identical data, MedFuse and MeTra achieve substantially lower performance (AUROCs of 0.667 and 0.641, respectively), highlighting the importance of model architecture in capturing these temporal patterns.

### Training on paired X-ray and clinical measurements data

Having investigated single modality training we combine two modalities, clinical measurements (C) and CXR images (X) as was done by MedFuse and MeTra as well.

#### MIMIC In-hospital mortality

The predictive performances of MeTra and ViTiMM can be improved by combining C and X. When trained on paired clinical measurements and CXR images MeTra achieves an AUROC of 0.859, MedFuse on the other hand only reaches 0.805. Our method, ViTiMM, outperforms both of them in terms of AUROC of 0.875 but also in AUPRC and balanced accuracy (see Table 2). In their paper Hayat et al. report an AUROC of 0.865 for training on clinical measurements and CXR images^29^. However, this was achieved when also training on unpaired samples, which we omit to achieve maximal comparibility. Additionally, our method also outperforms MedFuse trained on partial samples when only trained on paired samples.

#### MIMIC phenotyping

Similar to *In-hospital mortality* the performance of all methods can be enhanced by using both, clinical measurements and CXR images. MeTra achieves an AUROC of 0.712, MedFuse of 0.733. In their paper Hayat et al. again report a slightly higher AUROC of 0.770, which is achieved through training on the partial paired dataset^29^. However, with our method we again also outperform MedFuse’s partial training setting slightly with 0.778. AUPRC and balanced accuracy follow the same trends.

#### MIMIC decompensation

When leveraging both clinical data (*C*) and chest X-ray images (*X*) for decompensation prediction on the MIMIC-IV dataset, all models demonstrate improved predictive performance compared to their single-modality counterparts. For example, MMFusion achieves an AUROC of 0.715 in the multi-modal setting, representing a notable improvement over its best single-modality performance of 0.688 (X-ray only). ViTiMM exhibits the strongest performance among all evaluated methods, reaching an AUROC of 0.797 with *C* and *X* surpassing its already superior single-modality baseline of 0.781.

#### CDSL In-hospital mortality

For *in-hospital mortality* prediction on the CDSL dataset, MeTra and MedFuse demonstrate substantial performance gains over their uni-modal baselines in the multi-modal setting, achieving AUROCs of 0.724 and 0.690, respectively. In contrast, MMFusion exhibits a slight performance decrease to an AUROC of 0.905 compared to its single-modality performance, though it still outperforms both MeTra and MedFuse. ViTiMM maintains its superior performance with an AUROC of 0.987, matching its single-modality result and significantly outperforming all other methods.

### Extension to all modalities

To the best of our knowledge no method in the literature has so far investigated training on the four modalities, clinical measurements (C), medications (M), X-ray images (X), and ECG scans (E). Thus, we do not have a direct comparison to our method.

#### MIMIC in-hospital mortality

When feeding all modalities, clinical measurements, CXR images, medications, one ECG scan, as well as the corresponding demographics, CXR reports, ECG machine measurements, and patient’s diagnoses the predictive performance can significantly be improved. ViTiMM reaches an AUROC of 0.922 outperforming the two modality training across all methods. Also in terms of AUPRC (0.764) and balanced accuracy (0.847) training with all modalities represents the benchmark.

#### MIMIC phenotyping

For *phenotyping* the predictive performance of our method cannot be enhanced to a similar extend as for *In-hospital Mortality*. Still, we achieve an AUROC of 0.784 by combining clinical measurements, administered medications, CXR images, and ECG scans. The AUPRC is 0.549, balanced accuracy lies at 0.659. In contrast to the mortality prediction task we cannot feed the patient’s diagnoses as they shall be predicted from the input data.

#### MIMIC decompensation

While incorporating all four modalities generally enhances performance across other prediction tasks on the MIMIC dataset, decompensation prediction exhibits a marginal decrease when extending beyond the bi-modal setting, with ViTiMM’s AUROC declining slightly from 0.797 to 0.793. This suggests that the additional modalities may introduce noise rather than complementary information for this specific task. Nevertheless, ViTiMM maintains its superior performance, consistently outperforming all three comparison methods across both bi-modal and four-modal configurations.

#### CDSL in-hospital mortality

In the three-modal setting on the CDSL dataset, which lacks ECG data, ViTiMM maintains comparable AUROC performance (0.987) to the bi-modal configuration. However, examination of additional metrics reveals modest improvements, with AUPRC increasing from 0.933 to 0.934 and balanced accuracy rising from 0.937 to 0.958.

### Interpretability with attention visualization

**Fig. 6:**
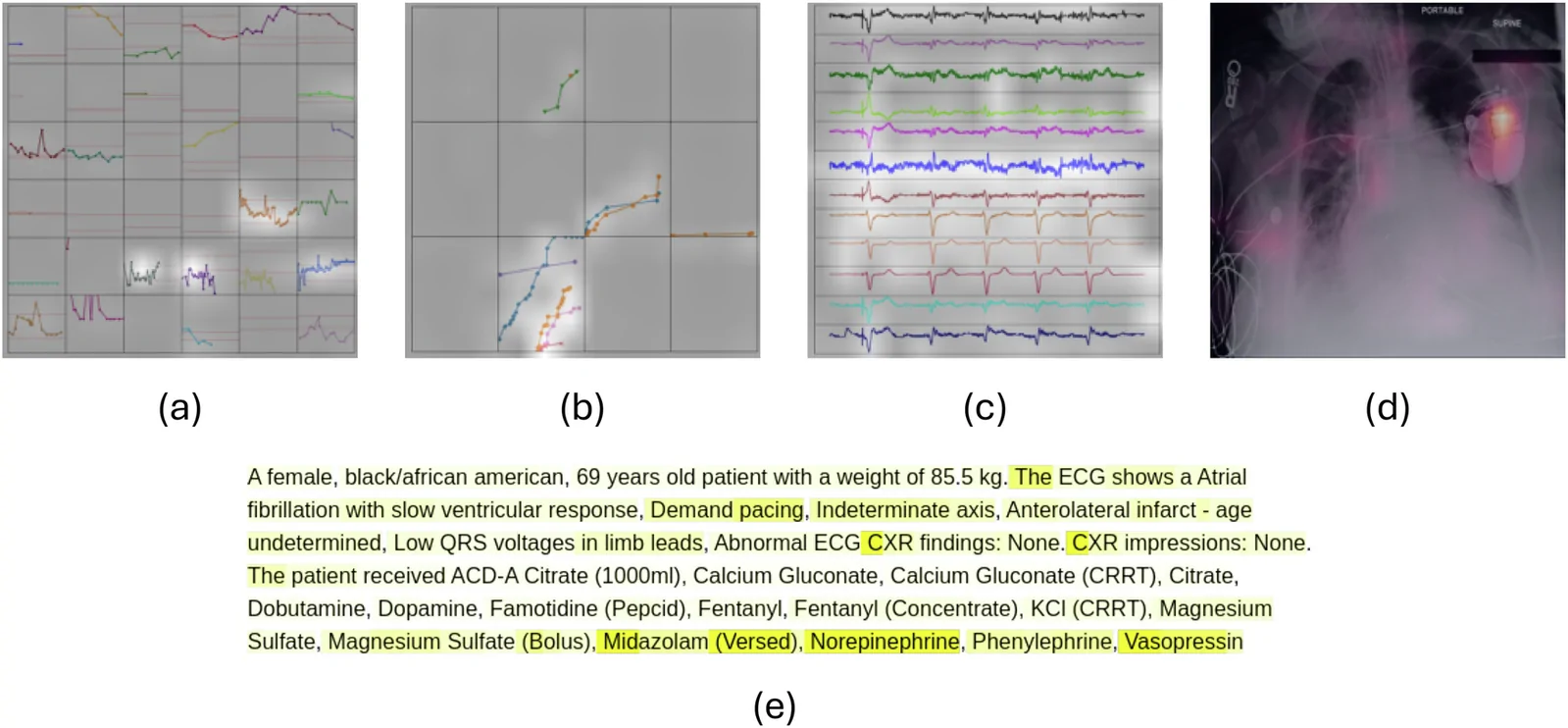
Interpretability with Visualizing Attention Maps for in-hospital mortality prediction for hadm_id = 30315583. The patient has died within the hospital stay, which the model rightfully predicted. The model clearly focuses on specific attributes in the clinical measurements (**a**) and medications (**b**). In contrast, the attention for ECG (**c**) appears more diffuse, suggesting that the model leverages a broader, more global context. For the CXR image (**d**), the attention is particularly concentrated on the implanted device. In the additional information given as text to the model the attention focuses especially on the administered medications (**e**). More examples can be found in the Supplemental Material.

Transformer architectures use the attention mechanism for trading of local and global contexts in vision tasks^17^. When using the conventional ViT the importance of input features can be visualized by overlaying the attention weights of the classification. The same procedure can be conducted for the text input. Our interpretability approach builds directly on the work of Li et al.^23^, who demonstrated that representing time series data in a structured, grid-like visual format enables vision transformers to produce meaningful attention patterns without explicit guidance. The structured visual encoding itself serves as implicit guidance: by plotting variable trajectories over time, temporal trends and clinically relevant patterns become spatially localized within the image, allowing the attention mechanism to focus on informative regions in a principled way. The patient, whose input data is shown in Fig. 6, has not survived his hospital stay. The model specifically focuses on specific features in the clinical measurements data, namely Respiratory Rate (from bottom left row 2, column 6), Non Invasive Blood Pressure Diastolic (2,4), Non Invasive Blood Pressure Diastolic (2,3), and Heart Rate (3,5) (Fig. 6a with corresponding field names in Supplementary Fig. 1). This attention pattern is clinically meaningful, as these vital signs represent core hemodynamic and respiratory parameters that are critical indicators of patient stability in the ICU^46,55^. Deteriorating respiratory rate and blood pressure values are established predictors of poor clinical outcomes and impending mortality in critically ill patients. In the medication inputs the most weight is put on Vasopressors and Inotropes (1,2), more precisely Norephinephrine, Phenylephrine, and Dobutamine, but the model attends to all administered medications to a small degree (Fig. 6b with corresponding field names in Supplementary Fig. 2). The model’s focus on vasopressors and inotropes is particularly significant, as these medications are typically administered in cases of severe hemodynamic instability, shock, or cardiovascular collapse, which are conditions strongly associated with increased mortality risk in ICU patients^43^. The attention on the ECG scan is diffuse, the focus rather seems to lie on the overall interaction of all leads. However, the largest attention lies on lead aVL (blue in Fig. 6c). The relatively diffuse ECG attention pattern suggests that individual ECG abnormalities may be less discriminative for mortality prediction in this patient cohort, presenting an opportunity for future model improvements through enhanced ECG-specific feature engineering or specialized cardiac rhythm analysis. In the CXR image specific focus lies on the implanted device (Fig. 6d). The model’s attention to the implanted pacemaker is clinically relevant, as the presence of cardiac devices often indicates underlying severe cardiovascular disease and represents a significant comorbidity factor that can influence mortality risk in ICU patients. In the text input the model directs specific attention to the administered medications and the implanted pacemaker. General attention lies on the patient demographics and ECG findings. While no findings and impressions are present in the CXR report, the attention peaks in this part indicating general importance if information exists. Further attention visualizations for both classes in the *In-hospital mortality* task can be found in Supplementary Figs. 5 and 6. The observed attention patterns, where the model consistently attends to clinically established predictors across modalities, are consistent with results reported by Li et al.^23^ under a comparable evaluation setup, further supporting the validity of interpreting attention weights in this visual encoding framework.

## Discussion

We present a method that can learn from arbitrary modalities by employing vision transformer only by representing the respective data in image format as an extension to ViTST^23^. We demonstrate that by representing any modality as an image, in a manner broadly analogous to how humans interpret data, multi-modal training can be achieved with minimal effort. This is because vision transformers are inherently capable of capturing the context of data in a way that resembles human perception. This approach simplifies the training process significantly, it is broken down to *visual prompt engineering*, which can be performed with little coding knowledge. We hope this will lower the barrier of multi-modal training immensely in the future. Our model not only simplifies multi-modal training in medicine but also shows superior performance on the benchmark tasks of in-hospital mortality and decompensation prediction as well as phenotyping^38^ compared to approaches in the literature^28–30^. To the best of our knowledge, we are the first to train one model across the modalities of clinical measurements, CXR images, medications, and ECG scans as well as further patient metadata on the MIMIC-IV^22,35–37^ and CDSL dataset^33^.

Humans are able to quickly grasp information and trends from visual prepared graphical data. Vision transformer seem to be able to capture information in a similar manner also observing interactions between different variables. The approach is especially beneficial for irregularly sampled time series data^23^. Most approaches need to explicitly model the time dimension by e.g., partitioning the data in fixed time intervals^56,57^ or use dedicated architectures such as LSTMs or graph neural networks^26,58,59^. However, modeling the time dimension might lead to loss of information dependent on e.g., the sampling rate when partitioning observations. On the other side, when representing the data as line graphs observations can be explicitly marked and missing intervals interpolated in-between. The model is made aware of the missing information in this area, but is presented a hint for possible values in the interval. The possible information loss is then dependent on the resolution (see below). The advantage is emphasized when only comparing the results for clinical measurements (C) across the three methods (MMFusion^30^, MeTra^28^, MedFuse^29^, and ViTiMM) in Table 2, where our method obtains the best performance across all metrics. This effect is particularly pronounced on the CDSL dataset, where ViTiMM achieves an exceptional AUROC of 0.986 using clinical measurements alone. We attribute this superior performance to the model’s enhanced ability to capture global temporal patterns, as patients who did not survive their hospital stay exhibit substantially greater variability in their vital signs throughout the monitoring period (see Fig. 5). In MeTra for each clinical parameter in question the mean over the time period is taken; in MedFuse and MMFusion all measurements must be discretized and harmonized to similar time points across variables.

Our results confirm that multi-modal training is beneficial for all investigated methods for all tasks under consideration. The multi-modal approach enables the extraction of complementary information from the used modalities. Our approach, ViTiMM, outperforms the three comparative approaches (MeTra^28^ and MedFuse^29^) with the same data sources, clinical measurements and CXR images. We attribute this to potential information loss in the time series data caused by averaging the variables over the respective timeframe (see above).

The key difference between ViTiMM and other approaches in the literature is its straightforward extensibility to other modalities by either representing the data as an image such as e.g., line graphs or providing the data as unstructured text. Thus, we are also able to feed the medications of each patient despite the high missing ratios (see Supplementary Table 6), the ECG scan, and diagnoses of the patients without altering our encoders. While our method already achieves the best results compared to MeTra and MedFuse with only clinical measurements and CXR images, the additional modalities lead to even better performances. Extension of MMFusion, MeTra, and MedFuse to other modalities is not straightforward, as each modality must be separately modeled and, thus, receive a different encoder than used currently in their methods. This would alter the presented methods drastically, which might not align with the intentions of the authors. In general, especially medications would require much modeling due to high missing ratios and the different dosages of different solutions of the active ingredients.

While visual transformation of time series data offers advantages for leveraging pre-trained vision models, it necessarily introduces information loss through temporal resampling that needs careful analysis.

Let ${{{\mathcal{S}}}}(t)$ for *t* ∈ [0, *T*] be a normalized time series, rasterized to resolution *W* × *H*. In the temporal dimension, plotting *N* samples onto *W* pixels effectively aggregates *N*/*W* samples per pixel, yielding an effective sampling rate of *W*/*T* where *T* = 48 h. The Nyquist frequency is (*W*/2*T*), beyond which aliasing may occur. F or standard resolutions, this yields Nyquist frequencies of 2.33 samples/hour (224 × 224) and 4.00 samples/hour (384 × 384). Our empirical analysis across 33 clinical variables reveals that while some variables exhibit maximum sampling frequencies up to 60 samples/hour (Supplementary Table 13), these represent outlier measurements. Critically, the mean sampling frequencies across all variables remain well below the Nyquist threshold: 1.22 samples/hour overall, with 95th percentiles typically under 15 samples/hour for most variables. This indicates that while peak sampling rates may theoretically suffer from aliasing, the majority of clinical measurements operate within safe frequency ranges for visual transformation.

It is important to note that temporal resampling and quantization are fundamental challenges in any time series analysis methodology, not unique to visual transformation. Traditional approaches to irregularly sampled clinical data routinely employ interpolation, resampling, and discretization techniques that introduce similar information transformations. For instance, standard time series preprocessing typically involves resampling to fixed intervals, missing value imputation, and normalization.

Our experiments demonstrate that higher resolution (384 × 384) consistently improves performance over standard resolution (224 × 224), supporting the hypothesis that information preservation through enhanced visual fidelity contributes meaningfully to model performance.

Careful consideration must be made regarding possible confounding variables in the input modalities. For example, certain of our considered medications, particularly opiods and sedatives, are commonly administered before death. To assess their impact on the accuracy of predictions, we analyzed the model outputs for those using medications as input (all modalities and medications only). We compared the performance across two subgroups: patients who received opiods and/or sedatives (group 1) vs. those who did not (group 2). Comparing AUROC values, we observed a slight increase in performance for group 1 (0.942 vs. 0.930 in the all modality setting and 0.768 vs. 0.751 for medications only). However, a DeLong test found these differences not statistically significant (*p* = 0.546, *p* = 0.580)^60^.

The superiority of using multiple modalities for training due to their complementary information has been proven multiple times^9,28–30^. However, public datasets with multiple modalities are scarce. Especially in medicine, the literature focuses on the MIMIC-IV dataset^22,28,29,61,62^. The MIMIC-IV is a role model for an open source multi-modal dataset covering several modalities namely clinical measurements, medications, CXR images, ECG scans, and recently also echocardiography (ECHO) data^63^. Benchmark tasks are defined with a extraction pipeline establishing comparability between studies^38^. However, some discrepancies still occur when paired or partial samples across modalities are used^28,29^. Further public multi-modal benchmark datasets are needed to examine the generalizability of our and other methods. In future work we also plan to incorporate echocardiography data into our pipeline, which technically is straightforward as they are naturally represented as images. However, echocardiography data has a very large intra-patient variance due to multiple views, which are not explicitly labeled or identified in the dataset.

Additionally, we selected the CDSL dataset, also available through PhysioNet^33^, as an additional multi-modal evaluation dataset to assess generalizability beyond MIMIC-IV. However, unlike MIMIC-IV, CDSL lacks established benchmark tasks and standardized evaluation protocols. To enable meaningful comparison, we adapted the in-hospital mortality prediction task from MIMIC-IV to the CDSL dataset, maintaining consistent outcome definitions and evaluation metrics across both datasets.

## Conclusion

We presented ViTiMM, a method that reduces multi-modal medical data integration to visual prompt engineering by representing each modality as an image and training conventional vision transformers. Our results on the MIMIC-IV and CDSL datasets demonstrate that line graph representations of irregularly sampled time series preserve sufficient information to outperform dedicated time series architectures across in-hospital mortality, phenotyping, and decompensation prediction. By unifying the feature extraction pipeline across modalities, our approach enables seamless extension to additional data sources such as medications and ECG scans without architectural changes, a capability no competing method currently offers. This substantially lowers the barrier for clinicians and non-technical researchers to leverage multi-modal data, as the primary design choice reduces to how best to visualize each modality rather than how to engineer modality-specific encoders. We believe this paradigm of visual prompt engineering can generalize beyond the modalities and tasks considered here, for instance to echocardiography or waveform data from bedside monitors. In future work, we plan to explore more expressive fusion strategies such as cross-attention mechanisms between modality-specific representations, which may capture inter-modal dependencies more explicitly and further improve predictive performance.

## Supplementary information


Transparent Peer Review file
Supplementary Material


## Data Availability

All data is publicly available from the MIMIC database^22,34^ on PhysioNet (MIMICIV: https://physionet.org/content/mimiciv/3.1, MIMIC-CXR-JPG: https://physionet.org/content/mimic-cxr-jpg/2.0.0, and MIMIC-IV-ECG: https://physionet.org/content/mimic-iv-ecg/1.0). The COVID Data for Shared Learning (CDSL) dataset is available at https://physionet.org/content/covid-data-shared-learning/1.0.0/. The code to extract the chest radiographs and corresponding clinical parameters can be found in the GitHub repository linked in the code availability section.

## Code availability

Following the FAIR criteria (findability, accessibility, interoperability, and reusability) in scientific research all code used in this study will be made publicly available. The code for cohort creation, data preprocessing, i.e., plot generation, training, and evaluation is open source available at https://doi.org/10.5281/zenodo.20786553 and at https://github.com/Cardio-AI/ViTiMM.
