# Supplementary material for: Visual prompt engineering for multimodal and irregularly sampled medical data
Source: Commun Med (Lond). 2026 Sep 1;6:474. doi: 10.1038/s43856-026-01817-x (PMC13534428; doi:10.1038/s43856-026-01817-x)
Supplement: Supplementary file 1 [file 43856_2026_1817_MOESM1_ESM.pdf]

nature portfolio

Peer Review File

Visual Prompt Engineering for Multimodal and Irregularly Sampled Medical Data

Corresponding Author: Mr Malte Toelle

This file contains all reviewer reports in order by version, followed by all author rebuttals in order by version.

Version 0:

Reviewer comments:

Reviewer #1

(Remarks to the Author)
Summary: This paper introduces an approach for multimodal and irregularly sampled clinical data. It transforms diverse medical data types—including clinical measurements, medication records, X-ray images, and ECG scans—into standardized visual representations, so all these data can be processed by a unified vision transformer architecture. It demonstrates remarkable performance on in-hospital mortality prediction, achieving an AUROC of 0.922 when combining multiple modalities. The visual representation approach also offers enhanced interpretability.

Questions:
1. While image-based representation proves effective, I'm interested in understanding how this compares to other potential approaches. How does this approach compare to EHR into sequence-based representations? Could a specialized tokenizer (recent work like https://arxiv.org/pdf/2412.16178) potentially offer competitive performance? What's the advantage of visual representation.

2. What is the performance benchmark of current general Vision-Language Models (VLMs) such as Qwen-2.5-VL, O3, or Gemini-2.5-Pro on similar clinical prediction tasks?

3. Would initializing the vision encoder with a pre-trained Chest X-Ray (CXR) vision encoder potentially improve model performance?

4. How robust is the ViTiMM approach to different temporal scales, like patient records spanning extremely short periods (hours) / cover a long time range? Any normalization or adaptation strategies are employed to manage varying data densities and temporal variations?

5. [Optional] While the current study effectively demonstrates performance on in-hospital mortality prediction and phenotyping, the research would benefit from exploring a broader range of downstream prediction tasks, such as re-admission risk assessment.

Reviewer #2

(Remarks to the Author)
In this study, ViTiMM (Vision Transformer for irregular sampled Multi-modal Measurements) is proposed, an approach that simplifies multi-modal medical data modeling by visualizing all data types—including temporal measurements, interventions, and imaging—as unified image-like representations. By training a vision-text transformer on these visualizations, the authors reduced the complexity of traditional multimodal models that typically require specialized components for each data type. Despite the interesting nature of the study, although it is not a particularly new idea, there

are major concerns that need to be addressed:
- The introduction must be rewritten to reflect a clear problem definition, a review of relevant literature, and then the objectives and contributions. Currently, the introduction is lengthy and lacks coherence.
- The methodology section is missing! While there is a section after the “discussion” labeled as “method”, the writing lacks scientific rigor and has poor structure, making it difficult to follow.
- The proposed model structure is not properly explained. It is unclear why the model is only compared to two related studies and whether the comparisons are fair.
- The model’s performance in the single-modality setting seems suboptimal. Moreover, in multi-modality settings, the performance improvement seems counterintuitive, as one would expect reduction rather than improvement.
- The interpretability evaluation using pre-structured inputs seems questionable. It is unclear how the model can achieve meaningful focus without explicit guidance, especially with ~80% accuracy.
- The comparisons are insufficient and do not provide enough evidence for the model’s superiority.
- Some abbreviations are not explained, such as GPD or EHR.

Reviewer #3

(Remarks to the Author)
The article introduces a novel approach termed ViTiMM, specifically designed to simplify the integration and analysis of multimodal, irregularly sampled clinical data. By converting various patient data modalities, including clinical measurements, medication records, ECG scans, and X-ray images, into visual representations, the authors leverage the capabilities of vision transformers to unify data processing and significantly reduce modelling complexity. This technique, effectively a form of "visual prompt engineering" bypasses the traditional challenges associated with separately modelling each modality. Evaluations on the MIMIC-IV dataset demonstrate that ViTiMM consistently outperforms 2 baseline methods in critical predictive tasks such as in-hospital mortality prediction and clinical phenotyping. Additionally, ViTiMM offers enhanced interpretability via attention maps, providing insight into influential data regions contributing to model predictions. The authors advocate that their approach not only enhances predictive performance but also substantially lowers the entry barrier for developing advanced multimodal AI models in medicine, potentially enabling broader adoption through simplified, no-code solutions.

Strengths:

The paper's primary strength lies in its elegant simplicity: by translating diverse and irregularly sampled medical data into visual formats, the authors ingeniously simplify the complex task of multimodal integration into a single, cohesive vision transformer-based pipeline. This visual prompt engineering approach significantly reduces the barriers typically associated with modality-specific modelling, enabling the incorporation of numerous modalities, clinical measurements, medications, ECG, and imaging data, without substantial architectural changes. The robust evaluation on the widely-used MIMIC-IV dataset, demonstrating clear improvements over existing baseline methods, further reinforces the practical relevance and efficacy of this approach. Additionally, the explicit interpretability provided through attention map visualisations substantially enhances clinical trust and usability, positioning this method strongly for real-world clinical deployment.

Major concerns:

The major concerns or weaknesses of this paper primarily stem from its methodological assumptions and the potential limitations regarding generalisability. Firstly, while the visual transformation simplifies modality integration, it implicitly assumes that vision transformers can effectively capture nuanced temporal dynamics and subtle inter-modal interactions purely from visual prompts, an assumption not yet comprehensively validated across diverse clinical scenarios. Secondly, the model's performance gains are demonstrated exclusively on the MIMIC-IV dataset, raising questions about its external validity across different healthcare settings, clinical practices, or patient populations. Thirdly, representing medical data visually, particularly time series with significant missingness or irregular sampling, introduces risks of information distortion or loss, as subtle temporal details might be inadequately captured by simple line graphs. Most importantly, this method of working with clinical parameters or medications (through the use of line graphs) is novel. However, it unequivocally requires explicit benchmarking in order to demonstrate that treating such data in this manner is both acceptable and superior to employing the raw data in its original form. I would argue that the use of line graphs, as opposed to the raw data itself, merely increases complexity and fails to present any sound methodological or mathematical justification. The authors must provide a stronger rationale for employing the data in this manner, and they must also compare it against traditional approaches to working with raw data, which are already well established. Additionally, although attention visualisations enhance interpretability, they offer limited clinical insight into causal relationships, potentially misleading clinicians unfamiliar with transformer-based methods. Furthermore, while attention maps are helpful, providing additional quantitative metrics (e.g., attention entropy, feature importance scores, or statistical analyses of attended regions) would strengthen interpretability beyond qualitative insights alone. Additionally, the authors should consider using newer baselines, as the current ones are not from 2025 or even 2024. [14] is from 2022 and [27] is from 2023. For example (just ideas, might not be very related):

1. [This method outperforms MeTra[27]] Wang, Muyu, et al. "Robust multi-modal fusion architecture for medical data with knowledge distillation." Computer Methods and Programs in Biomedicine 260 (2025): 108568.

2. [This method outperforms MedFuse [14]] Liu, Xinlong, et al. "MDFormer: Transformer-Based Multimodal Fusion for Robust

Chest Disease Diagnosis." Electronics 14.10 (2025): 1926.

3. [A cutting-edge preprint from Microsoft Research] Lee, Ho Hin, et al. "Multi-Modal Mamba Modeling for Survival Prediction (M4Survive): Adapting Joint Foundation Model Representations." arXiv preprint arXiv:2503.10057 (2025).

4. Kumar, Sachin, Shivani Sharma, and Kassahun Tadesse Megra. "Transformer enabled multi-modal medical diagnosis for tuberculosis classification." Journal of Big Data 12.1 (2025): 5.

5. Wang, Muyu, et al. "Missing-modality enabled multi-modal fusion architecture for medical data." Journal of Biomedical Informatics 164 (2025): 104796.

Finally, the potential confounding effects of certain modalities (e.g., medications closely associated with patient outcomes like sedatives or opioids administered near death) necessitate scrutiny to avoid biased predictions or spurious correlations in clinical deployment.

Minor concerns:

Conducting more detailed ablation experiments could clarify the incremental benefit provided by each individual modality and help justify their inclusion more thoroughly. Reporting detailed computational resource requirements (FLOPs, training/inference time, GPU usage, environmental impact) would be valuable for practical adoption and reproducibility. Minor improvements in explicitly linking model insights (such as highlighted attention regions) to clinically meaningful indicators or standard clinical assessments would strengthen the clinical relevance and interpretability.

Version 1:

Reviewer comments:

Reviewer #1

(Remarks to the Author)
Thank you for addressing my previous concerns. I have no further questions or comments.

Reviewer #2

(Remarks to the Author)
I thank the authors for carefully considering the reviewers’ comments and for their efforts to improve the quality of their manuscript based on the feedback provided. However, I am not fully satisfied with the responses to some of the questions and concerns, as outlined below:

1- I appreciate the authors’ clarification; however, the response to Q3 remains not entirely satisfactory. Upon reviewing several recently published articles in this journal, I observed that all follow the conventional structure: 1. Introduction, 2. Materials and Methods, 3. Results, 4. Discussion, and 5. Conclusion. Given this established format, the justification provided does not appear consistent with the journal’s current publication standards. I therefore leave it to the Editor’s discretion to decide whether the manuscript’s present structure is acceptable.
2- In response to Q4: I could not locate the addition referred to as being made “to the model architecture section.” Please clarify where exactly these changes have been implemented, as this addition is not evident in the revised manuscript.
3- In response to Q5: The authors have provided an explanation of rather obvious points, while the main concern was to include supporting evidence or data within the manuscript. The current response does not adequately address this issue, as the paper still lacks the necessary evidence to substantiate the statements made.
4- In response to Q6: My previous concerns remain unresolved. The authors have again provided an explanation of rather obvious points, without supporting these claims with appropriate results or analyses. Therefore, the response does not satisfactorily address the issue raised.
5- Code Repository: Upon attempting to access the GitHub repository provided in the manuscript, it appears that the link is incorrect. The authors should verify and correct the repository link to ensure that readers can properly access the code and reproduce the results.
Although I previously recommended rejection of the manuscript, I acknowledge that the final decision after considering other reviewers’ opinions was to invite revision. While I recognize the authors’ efforts to address the comments, several of my major concerns remain unresolved, as detailed above. Based on these reasons, I still find the manuscript not suitable for publication in its current form. However, I will defer to the Editor’s judgment to determine whether the revisions made are sufficient for acceptance.

Version 2:

Reviewer comments:

Reviewer #2

(Remarks to the Author)
I thank the authors for revising the paper. Although many comments have been addressed, my request to add more results and more comparisons to prove the superiority of the model has not been met. However, my final decision is to reluctantly accept the paper and I hope that further independent investigations by readers of the paper will be conducted in the future.

Open Access This Peer Review File is licensed under a Creative Commons Attribution 4.0 International License, which permits use, sharing, adaptation, distribution and reproduction in any medium or format, as long as you give appropriate credit to the original author(s) and the source, provide a link to the Creative Commons license, and indicate if changes were made.
In cases where reviewers are anonymous, credit should be given to 'Anonymous Referee' and the source.
The images or other third party material in this Peer Review File are included in the article's Creative Commons license, unless indicated otherwise in a credit line to the material. If material is not included in the article's Creative Commons license and your intended use is not permitted by statutory regulation or exceeds the permitted use, you will need to obtain permission directly from the copyright holder.
To view a copy of this license, visit https://creativecommons.org/licenses/by/4.0/

Response to Reviews for Visual Prompt Engineering for Multimodal and Irregularly Sampled Medical Data

General Response

thank you for sending us the high-quality reviews for our manuscript Visual Prompt Engineering for Multimodal and Irregularly Sampled Medical Data in consideration for publication in Nature Communications Medicine. The valuable feedback from the reviewers greatly helped to further improve our paper. With this letter, you receive an updated version incorporating all issues raised with particular emphasis on extending the evaluation to an additional dataset and task, incorporating a further baseline method for comparison, and providing comprehensive discussion of the methodological assumptions and temporal robustness of our approach. Changes and additions in the revised manuscript are highlighted in red. In the following, we will address each reviewer comment point by point and hope to meet their expectations. We welcome an open discussion if any issues remain, or new questions arise.

On behalf of all authors, Malte Tölle

Response to Reviewer 1:

Q1 This paper introduces an approach for multimodal and irregularly sampled clinical data. It transforms diverse medical data types—including clinical measurements, medication records, X-ray images, and ECG scans—into standardized visual representations, so all these data can be processed by a unified vision transformer architecture. It demonstrates remarkable performance on in-hospital mortality prediction, achieving an AUROC of 0.922 when combining multiple modalities. The visual representation approach also offers enhanced interpretability.

A1 Thank you for your kind verdict. Your thoughtful remarks greatly helped us to improve our paper. We believe that this unified visual representation framework opens new possibilities for seamless multi-modal integration without sacrificing performance.

Q2 While image-based representation proves effective, I'm interested in understanding how this compares to other potential approaches. How does this approach compare to EHR into sequence-based representations? Could a specialized tokenizer (recent work like

https://arxiv.org/pdf/2412.16178) potentially offer competitive performance? What's the advantage of visual representation.

A2 Thank you for highlighting this relevant work. We are familiar with the referenced publication and acknowledge its innovative approach to EHR tokenization. However, we did not include it in our comparison as it focuses exclusively on single-modality EHR processing, whereas our work addresses the challenge of integrating multiple heterogeneous modalities (EHR, medical imaging, ECG signals).

Our chosen baselines (MeTra [5], MedFuse [3], and MMFusion [10]) already represent sequence-based approaches for multimodal clinical data, providing appropriate comparison to established methods that handle EHR data alongside other modalities.

The key advantage of our visual representation approach lies in its unified framework for multimodal integration. By transforming all modalities into a common visual format, we eliminate the need for modality-specific architectures and complex fusion mechanisms. This simplification enables the use of standard vision transformers while maintaining competitive performance, making multimodal clinical AI more accessible and implementable. Additionally, our approach naturally handles the varying temporal resolutions and data densities common in clinical settings through consistent visual encoding.

While specialized tokenizers for EHR data represent an important research direction, our visual representation strategy addresses the broader challenge of seamlessly integrating diverse clinical data types within a single, coherent framework.

Q3 What is the performance benchmark of current general Vision-Language Models (VLMs) such as Qwen-2.5-VL, O3, or Gemini-2.5-Pro on similar clinical prediction tasks?

A3 While comprehensive benchmarking of modern VLMs on clinical tasks would be valuable, such evaluation represents a substantial research project beyond our current scope. Thorough investigation of prompting strategies, data representation methods, and model behaviors would require extensive resources that diverge from our primary objective of demonstrating efficient fine-tuning approaches for vision transformers on consumer hardware.

To provide preliminary insight, we conducted a limited evaluation using Anthropic's Claude Sonnet 4 via their Model Context Protocol (MCP) server integration, which enabled automated pipeline processing of MIMIC-IV data on our local infrastructure. The implementation is available at https://gist.github.com/maltetoelle/b2839cfa4c48197aef44cc102758bede.

Our results support our core thesis: when provided only with raw input data and basic task context, the general-purpose LLM performed poorly on clinical risk prediction. Across 50 test patients, the model failed to correctly identify any cases as high-risk for in-hospital mortality, demonstrating the necessity of specialized fine-tuning for clinical prediction tasks.

Q4 Would initializing the vision encoder with a pre-trained Chest X-Ray (CXR) vision encoder potentially improve model performance?

A4 Thank you for pointing out this possible improvement. However, all our experiments were performed with the SWIN transformer pretrained on CheXpert dataset [4]. We apologize for not explicitly stating this in the original manuscript.

⇒ We have added the information in the architecture section.

Q4 How robust is the ViTiMM approach to different temporal scales, like patient records spanning extremely short periods (hours) / cover a long time range? Any normalization

or adaptation strategies are employed to manage varying data densities and temporal variations?

A4 Our primary evaluation follows established benchmarks using a standardized 48-hour window, ensuring fair comparison with baseline methods while avoiding extreme temporal distribution cases as defined in [2]. We employ value standardization to reduce outlier influence and mitigate temporal density variations.

To empirically assess temporal robustness, we conducted additional experiments on the COVID Data for Shared Learning (CDSL) dataset [9], which presents different temporal characteristics than MIMIC-IV. While CDSL contains similar modalities (laboratory measurements, vital signs, chest X-rays, and medications), it lacks ECG data and exhibits coarser temporal granularity with fewer measurements per day. This required adapting our temporal window to five days across all models.

Despite these substantial differences in temporal scale and data density, ViTiMM maintained superior performance for the task of in-hospital mortality prediction: achieving 0.987 AUROC compared to MeTra (0.724), MedFuse (0.690), and MMFusion (0.905). These results demonstrate that our approach generalizes effectively across varying temporal scales and measurement frequencies.

The consistent performance across datasets with different temporal characteristics suggests that ViTiMM's architecture effectively handles varying data densities through its adaptive attention mechanisms, though we acknowledge that performance depends on input resolution and potential information loss during temporal compression.

⇒ We added experiments on another method (MMFusion), another dataset (CDSL), and a paragraph in the discussion concerning different temporal scales and the induced information loss.

Q4 [Optional] While the current study effectively demonstrates performance on in-hospital mortality prediction and phenotyping, the research would benefit from exploring a broader range of downstream prediction tasks, such as re-admission risk assessment.

A4 We acknowledge the importance of evaluating across diverse clinical prediction tasks. In response to feedback, we have expanded our evaluation to include additional downstream tasks and datasets.

⇒ We incorporated the decompensation prediction task on MIMIC-IV, which requires predicting whether a patient will experience clinical deterioration during their hospital stay. Additionally, we validated our approach on the COVID Data for Shared Learning (CDSL) dataset [9] for in-hospital mortality prediction. While similar in objective to MIMIC-IV, CDSL presents distinct challenges with coarser temporal resolution requiring a 5-day prediction window instead of 48 hours. This evaluation simultaneously addresses concerns about temporal robustness and generalizability across different healthcare systems and patient populations.

Response to Reviewer 2:

Q1 In this study, ViTiMM (Vision Transformer for irregular sampled Multi-modal Measurements) is proposed, an approach that simplifies multi-modal medical data modeling by visualizing all data types—including temporal measurements, interventions, and imaging—as unified image-like representations. By training a vision-text transformer on these

visualizations, the authors reduced the complexity of traditional multimodal models that typically require specialized components for each data type. Despite the interesting nature of the study, although it is not a particularly new idea, there are major concerns that need to be addressed:

A1 Thank you for the constructive feedback, which has significantly improved our manuscript. We acknowledge that data-to-image representation, particularly for time series, is a known technique in machine learning.

Our contribution extends this concept to the multi-modal clinical domain, where we demonstrate that heterogeneous data types (temporal measurements, medical interventions, and imaging data) can be effectively unified through visual representations while preserving cross-modal contextual relationships. This unified approach enables a vision transformer architecture to capture complex interactions between modalities that traditionally require specialized fusion mechanisms.

The practical significance lies in democratizing multi-modal clinical AI development. By reducing the implementation complexity to creating appropriate visual representations of each modality, our approach makes sophisticated multi-modal modeling accessible to researchers and practitioners without requiring expertise in complex architectural design. This "low-code" paradigm could accelerate the adoption of multimodal approaches in clinical settings where technical resources may be limited.

We believe our empirical validation across multiple datasets and tasks demonstrates that this simplified approach can achieve competitive performance while offering significant advantages in terms of implementation simplicity and generalizability.

Q2 The introduction must be rewritten to reflect a clear problem definition, a review of relevant literature, and then the objectives and contributions. Currently, the introduction is lengthy and lacks coherence.

A2 Thank you for your comment.

⇒ We have restructured the introduction to provide clearer distinction between problem motivation and our specific contributions. The revised introduction now follows a more streamlined organization: (1) Clinical problem definition and motivation in ICU settings, (2) Technical challenges in multimodal healthcare data, (3) Review of existing approaches and their limitations, and (4) Our specific objectives and contributions.

Q3 The methodology section is missing! While there is a section after the “discussion” labeled as “method”, the writing lacks scientific rigor and has poor structure, making it difficult to follow.

A3 Thank you for your feedback regarding the structure of the manuscript. We acknowledge that the placement and presentation of the methodology may not align with your expectations. However, we have followed the formatting guidelines provided by Nature (available at https://www.nature.com/nature/for-authors/formatting-guide), which suggest including methodological details after the discussion section. We appreciate your understanding and hope that the rationale behind this structure is clear in the context of submitting to a Nature Portfolio journal.

Q4 The proposed model structure is not properly explained. It is unclear why the model is only compared to two related studies and whether the comparisons are fair.

A4 We appreciate this feedback and acknowledge that our model architecture description could be clearer. Our approach deliberately employs established architectures to isolate and evaluate the core contribution: efficient multimodal data preprocessing and representation. ViTiMM utilizes the SWIN transformer [8] for visual processing and RoBERTa [7] for textual features, following the architectural framework of ViTST [6]. Features extracted from each modality are concatenated and passed through a classification layer. This straightforward fusion strategy intentionally minimizes architectural complexity to highlight our primary innovation: transforming heterogeneous clinical data into a unified visual representation that enables seamless multimodal integration.

Regarding comparison fairness, we selected baseline methods that represent the current state-of-the-art in multimodal clinical prediction. We have now expanded our evaluation to include additional comparison methods (MMFusion, as noted in response to R3) and validated across multiple datasets and tasks to ensure comprehensive assessment. All comparisons use identical data preprocessing, evaluation metrics, and experimental protocols to ensure fair evaluation.

The architectural simplicity is intentional, our goal is to demonstrate that effective multimodal clinical prediction can be achieved through innovative data representation rather than complex model architectures, making our approach more accessible and practically implementable.

⇒ We have added a paragraph to the model architecture section describing our intended multi-modal training procedure more formal.

Q5 The model's performance in the single-modality setting seems suboptimal. Moreover, in multi-modality settings, the performance improvement seems counterintuitive, as one would expect reduction rather than improvement.

A5 Thank you for your thoughtful comment and we understand your concern. However, we believe the results align with a central principle in machine learning: providing a model with more informative and complementary data (especially from distinct modalities) can enhance its predictive performance. In medical applications, different modalities often capture fundamentally different aspects of a patient's condition. For instance, while an X-ray image might reveal structural abnormalities (e.g., pulmonary edema), clinical measurements such as heart rate or glucose levels provide physiological insights not visible in imaging data. Our contribution lies in designing a unified framework that simplifies multi-modal integration by converting all data types like representations. This not only enables the use of standard vision architectures but also facilitates access for non-programmers to analyze heterogeneous medical data more effectively.

We hope this clarifies the rationale behind the observed performance trends and the design choices of our method.

Q6 The interpretability evaluation using pre-structured inputs seems questionable. It is unclear how the model can achieve meaningful focus without explicit guidance, especially with 80% accuracy.

A6 We appreciate the reviewer's concern. The guidance provided to the model stems from our visual prompt engineering strategy. Specifically, we present input data in a way that mimics human cognitive processing by visually encoding temporal trends and patterns into an image format. By plotting variable trajectories over time, we make temporal dependencies and trends directly observable, even without explicitly labeling them. This allows the model

to learn from the structured visual contexts, similar to how humans intuitively interpret plotted time series. Our aim was to explore whether neural networks, like humans, benefit from such representations when compared to raw tabular data. Our results suggest that this visual encoding is an effective way to present information, especially in cases of irregularly sampled time series. Additionally, this paradigm can be extended to other modalities (e.g., representing ECG signals as plots) enabling a unified image-based approach across data types. This visual strategy also lowers the technical barrier for domain experts such as physicians. Instead of implementing complex preprocessing pipelines per modality, they only need to convert their data into intuitive visual formats, which facilitates both interpretability and accessibility for non-programmers.

Q7 The comparisons are insufficient and do not provide enough evidence for the model's superiority.

A7 Thank you for your critical observation. We acknowledge that our initial evaluation could be more comprehensive. In response to this and similar feedback from other reviewers, we have significantly expanded our experimental validation.

⇒ We have included additional comparison methods, specifically MMFusion [10] alongside the original MeTra [5] and MedFuse [3] baselines, providing a broader spectrum of state-of-the-art multimodal approaches for comparison. Furthermore, we have expanded our evaluation to include additional benchmark tasks (decompensation prediction) and validated our approach on an independent dataset (CDSL [9]). Across all evaluations, ViTiMM consistently demonstrates competitive or superior performance compared to all baseline methods.

However, we emphasize that our primary contribution extends beyond performance metrics alone. Our approach demonstrates the feasibility of unified multimodal clinical prediction through innovative data representation. The key advantage lies in its simplicity and generalizability: by transforming all modalities into visual representations, we enable the use of standard vision transformer architectures without requiring complex modality-specific designs. This significantly reduces implementation complexity while maintaining competitive performance, making multimodal clinical AI more accessible to practitioners and researchers.

Q8 Some abbreviations are not explained, such as GPD or EHR.

A8 Thank you for this observation.

⇒ We have reviewed the manuscript and added definitions for all previously undefined abbreviations, including GDP (gross domestic product) and EHR (electronic health records), to improve readability and accessibility for readers across different domains.

Response to Reviewer 3:

Q1 The article introduces a novel approach termed ViTiMM, specifically designed to simplify the integration and analysis of multimodal, irregularly sampled clinical data. By converting various patient data modalities, including clinical measurements, medication records, ECG scans, and X-ray images, into visual representations, the authors leverage the capabilities of vision transformers to unify data processing and significantly reduce modelling complexity. This technique, effectively a form of "visual prompt engineering" bypasses the traditional

challenges associated with separately modelling each modality. Evaluations on the MIMIC-IV dataset demonstrate that ViTiMM consistently outperforms 2 baseline methods in critical predictive tasks such as in-hospital mortality prediction and clinical phenotyping. Additionally, ViTiMM offers enhanced interpretability via attention maps, providing insight into influential data regions contributing to model predictions. The authors advocate that their approach not only enhances predictive performance but also substantially lowers the entry barrier for developing advanced multimodal AI models in medicine, potentially enabling broader adoption through simplified, no-code solutions.

Strengths:

The paper's primary strength lies in its elegant simplicity: by translating diverse and irregularly sampled medical data into visual formats, the authors ingeniously simplify the complex task of multimodal integration into a single, cohesive vision transformer-based pipeline. This visual prompt engineering approach significantly reduces the barriers typically associated with modality-specific modelling, enabling the incorporation of numerous modalities, clinical measurements, medications, ECG, and imaging data, without substantial architectural changes. The robust evaluation on the widely-used MIMIC-IV dataset, demonstrating clear improvements over existing baseline methods, further reinforces the practical relevance and efficacy of this approach. Additionally, the explicit interpretability provided through attention map visualisations substantially enhances clinical trust and usability, positioning this method strongly for real-world clinical deployment.

A1 Thank you for your thoughtful review and for highlighting the key contributions of our work. We appreciate your recognition of ViTiMM's core innovation: the elegant simplification of multimodal integration through visual representation of diverse clinical data types.

Your summary accurately captures our primary objective: to reduce the complexity traditionally associated with handling heterogeneous medical data modalities by leveraging vision transformers in a unified framework. We are particularly pleased that you acknowledge both the practical performance gains demonstrated on MIMIC-IV and the enhanced interpretability through attention visualizations, as these were central design goals aimed at facilitating real-world clinical adoption.

We value the constructive feedback you've provided regarding the study's limitations and will address each of your concerns systematically in the following responses.

Q2 The major concerns or weaknesses of this paper primarily stem from its methodological assumptions and the potential limitations regarding generalisability. Firstly, while the visual transformation simplifies modality integration, it implicitly assumes that vision transformers can effectively capture nuanced temporal dynamics and subtle inter-modal interactions purely from visual prompts, an assumption not yet comprehensively validated across diverse clinical scenarios.

A2 Thank you for raising this critical concern about methodological validation, which is indeed fundamental for clinical applications. We acknowledge that our assumption regarding vision transformers' ability to capture temporal dynamics and inter-modal interactions through visual representations requires more comprehensive validation across diverse clinical contexts.

We recognize that our current study represents an initial exploration of this approach rather than a definitive solution to multimodal clinical AI. The scarcity of openly available multimodal medical datasets significantly constrains comprehensive validation efforts.

⇒ To address your concerns about validation scope and baseline currency, we have expanded our evaluation in several ways:

Extended Task Validation: We incorporated additional experiments on decompensation prediction using MIMIC-IV, broadening the range of clinical scenarios evaluated.

Cross-Dataset Validation: We added experiments on the COVID Data for Shared Learning (CDSL) [9] dataset for in-hospital mortality prediction, providing evidence of generalizability beyond MIMIC-IV.

Updated Baseline Comparisons: We included Missing-modality enabled multi-modal fusion (MMFusion) [10], a recently published method from 2025, alongside the original MedFuse and MeTra baselines to ensure more contemporary comparisons.

Temporal Granularity Assessment: The CDSL dataset presented an opportunity to evaluate our approach under different temporal scales, as data is recorded in days rather than hours. We adapted our methodology to use a 5-day prediction window, demonstrating flexibility across varying clinical time frames.

While these additions strengthen our evaluation, we acknowledge that comprehensive validation across the full spectrum of clinical scenarios remains an ongoing challenge for the field. Our work aims to establish a foundation for this visual transformation approach, with the understanding that broader validation will require collaborative efforts as more diverse multimodal clinical datasets become available.

Q3 Secondly, the model's performance gains are demonstrated exclusively on the MIMIC-IV dataset, raising questions about its external validity across different healthcare settings, clinical practices, or patient populations.

A3 Thank you for highlighting this important limitation regarding external validity. We acknowledge that demonstrating generalizability across diverse healthcare settings is crucial for establishing the robustness of our approach.

Our initial choice of MIMIC-IV was motivated by its unique position as the most comprehensive openly available multimodal clinical dataset with established benchmark tasks that enabled fair comparisons with existing methods like MedFuse and MeTra. However, we recognize the critical need to evaluate external validity.

⇒ To address your concerns about generalizability, we have expanded our evaluation beyond MIMIC-IV in several key ways listed above.

Q4 Thirdly, representing medical data visually, particularly time series with significant missingness or irregular sampling, introduces risks of information distortion or loss, as subtle temporal details might be inadequately captured by simple line graphs. Most importantly, this method of working with clinical parameters or medications (through the use of line graphs) is novel. However, it unequivocally requires explicit benchmarking in order to demonstrate that treating such data in this manner is both acceptable and superior to employing the raw data in its original form. I would argue that the use of line graphs, as opposed to the raw data itself, merely increases complexity and fails to present any sound methodological or mathematical justification. The authors must provide a stronger rationale for employing the data in this manner, and they must also compare it against traditional approaches to working with raw data, which are already well established.

A4 Thank you for pointing out the missing mathematical justification and the critical need to validate our visual transformation approach against traditional raw data methods. We acknowledge that representing clinical data as images constitutes a novel methodological

choice that requires rigorous theoretical foundation and empirical validation. We understand our paper as introducing this visual transformation method for multimodal data integration in medicine.

⇒ To address your concerns, we have added mathematical clarification and quantitative analysis of information preservation.

Mathematical Framework: Following the notation from [6], let $D = \{(C_i, M_i, X_i, E_i, T_i, y_i) | i = 1, ..., N\}$ denote our multimodal dataset containing N samples, where C represents clinical measurements, M medications, X X-ray images, E ECG data, and T text data.

For each irregularly sampled time series S_i, observations for variable d are given by sequences of tuples $[(t_1^d, v_1^d), (t_2^d, v_2^d), \ldots, (t_{n_d}^d, v_{n_d}^d)]$, where n_d is the number of observations. Our framework comprises: (1) a transformation function converting time series S_i into image x_i, and (2) a vision transformer classifier predicting label $\hat{y}_i$.

Given a set of time series line graphs $G_i = g_1, g_2, ..., g_D$ for a time series S_i, we arrange them in a single image x_i using a pre-defined grid layout. We adopt a square grid by default. Specifically, we arrange the D time series line graphs in a grid of size $l \times l$ if $l \times (l - 1) < D \leq l \times l$, and a grid of size $l \times (l + 1)$ if $l \times l < D \leq l \times (l + 1)$.

Information Loss Quantification: We provide comprehensive mathematical analysis of information preservation during visual transformation. Let $S(t)$ for $t \in [0, T]$ be a normalized time series with values in [0, 1], rasterized to resolution $W \times H$.

In the temporal dimension, plotting N samples onto W pixels effectively aggregates N/W samples per pixel, yielding an effective sampling rate of W/T where $T = 48$ hours. The Nyquist frequency is $(W/2T)$, beyond which aliasing may occur. For standard resolutions, this yields Nyquist frequencies of 2.33 samples/hour (224×224) and 4.00 samples/hour (384×384).

Our empirical analysis across 33 clinical variables reveals that while some variables exhibit maximum sampling frequencies up to 60 samples/hour (Table 1), these represent outlier measurements. Critically, the mean sampling frequencies across all variables remain well below the Nyquist threshold: 1.22 samples/hour overall, with 95th percentiles typically under 15 samples/hour for most variables. This indicates that while peak sampling rates may theoretically suffer from aliasing, the majority of clinical measurements operate within safe frequency ranges for visual transformation.

It is important to note that temporal resampling and quantization are fundamental challenges in any time series analysis methodology, not unique to visual transformation. Traditional approaches to irregularly sampled clinical data routinely employ interpolation, resampling, and discretization techniques that introduce similar information transformations. For instance, standard time series preprocessing typically involves resampling to fixed intervals, missing value imputation, and normalization—all of which modify the original signal characteristics.

Our experiments demonstrate that higher resolution (384×384) consistently improves performance over standard resolution (224×224), supporting the hypothesis that information preservation through enhanced visual fidelity contributes meaningfully to model performance. This resolution-performance relationship provides empirical evidence that the visual transformation captures clinically relevant information patterns.

Comparison with Raw Data Methods: We have systematically compared our visual transformation approach against established traditional methods including MeTra, MedFuse, and MMFusion. We acknowledge the critical need for direct comparison against traditional raw data approaches. However, establishing fair comparisons is challenging due to the heterogeneous nature of our multimodal data and the lack of standardized baselines handling all modalities simultaneously. Our current baselines (MedFuse, MeTra, MMFusion) represent the closest available comparisons using similar multimodal approaches.

The fundamental contribution of our work lies not in replacing traditional time series analysis, but in enabling seamless integration of multiple data modalities through a unified visual framework.

The current work establishes the feasibility and initial promise of visual transformation, with quantifiable information loss analysis demonstrating that clinical relevance is preserved for the majority of measurements.

Q5 Additionally, although attention visualisations enhance interpretability, they offer limited clinical insight into causal relationships, potentially misleading clinicians unfamiliar with transformer-based methods. Furthermore, while attention maps are helpful, providing additional quantitative metrics (e.g., attention entropy, feature importance scores, or statistical analyses of attended regions) would strengthen interpretability beyond qualitative insights alone.

A5 Attention entropy measures how widely attention distributes across sequence tokens. Our analysis for the task of predicting in-hospital mortality on the MIMIC-IV dataset reveals modality-specific patterns aligned with clinical reasoning: clinical measurements show highly focused attention (entropy = 0.66 ± 0.40), indicating selective focus on key biomarkers; text demonstrates moderate focus (entropy = 1.92 ± 0.45) on diagnostic terminology; X-rays exhibit variable spatial attention (entropy = 2.81 ± 0.91) with high variance reflecting pathology-dependent focus; medications show moderate distribution (entropy = 3.04 ± 0.72) for drug interaction analysis; and ECG displays appropriately distributed temporal attention (entropy = 4.61 ± 0.43). The significant entropy variation across modalities (0.66-4.61) validates that our model learns data-type-specific attention strategies rather than uniform patterns, with low entropy for discrete features (clinical measurements) and higher entropy for complex relationships (temporal ECG patterns, spatial radiological findings). This quantitative evidence demonstrates clinically meaningful attention beyond qualitative visualizations.

Q6 Additionally, the authors should consider using newer baselines, as the current ones are not from 2025 or even 2024. [14] is from 2022 and [27] is from 2023. For example (just ideas, might not be very related):

1. [This method outperforms MeTra[27]] Wang, Muyu, et al. "Robust multi-modal fusion architecture for medical data with knowledge distillation." Computer Methods and Programs in Biomedicine 260 (2025): 108568.

2. [This method outperforms MedFuse [14]] Liu, Xinlong, et al. "MDFormer: Transformer-Based Multimodal Fusion for Robust Chest Disease Diagnosis." Electronics 14.10 (2025): 1926.

3. [A cutting-edge preprint from Microsoft Research] Lee, Ho Hin, et al. "Multi-Modal Mamba Modeling for Survival Prediction (M4Survive): Adapting Joint Foundation Model Representations." arXiv preprint arXiv:2503.10057 (2025).

4. Kumar, Sachin, Shivani Sharma, and Kassahun Tadesse Megra. "Transformer enabled multi-modal medical diagnosis for tuberculosis classification." Journal of Big Data 12.1 (2025): 5.

5. Wang, Muyu, et al. "Missing-modality enabled multi-modal fusion architecture for medical data." Journal of Biomedical Informatics 164 (2025): 104796.

A6 Thank you for highlighting the importance of comparing against more recent baseline methods and for providing these valuable suggestions. We acknowledge that using contemporary baselines strengthens the validity of our evaluation and better positions our work within the current research landscape.

Our initial selection of MedFuse [3] and MeTra [5] was primarily motivated by their direct applicability to the exact same tasks and datasets we evaluate, enabling fair and meaningful comparisons. However, we recognize the value of including more recent methods to demonstrate our approach's competitiveness against state-of-the-art techniques.

⇒ Following your recommendations, we have incorporated the Missing-modality enabled multi-modal fusion (MMFusion) method by Wang et al. [10] as an additional baseline. This selection was guided by several practical considerations:

Code Availability: MMFusion provided open-source implementation, enabling reproducible comparisons under identical experimental conditions.

Task Compatibility: The method addresses similar multimodal fusion challenges in clinical settings, making it a relevant comparison point.

Recent Publication: As a 2025 publication, it represents current state-of-the-art approaches in multimodal medical data fusion.

Regarding the other excellent suggestions, we encountered practical limitations that prevented their inclusion:

The Microsoft Research M4Survive method, while promising, requires pre-trained foundation models for all modalities. Unfortunately, such foundation models are not yet available for the clinical time series data and medication records present in MIMIC-IV, limiting our ability to implement a fair comparison. The other methods either lacked available implementations or addressed different clinical tasks, making direct comparison challenging within our experimental framework.

Q7 Finally, the potential confounding effects of certain modalities (e.g., medications closely associated with patient outcomes like sedatives or opioids administered near death) necessitate scrutiny to avoid biased predictions or spurious correlations in clinical deployment.

A7 Indeed, some of the medications we considered, particularly opiods and sedatives, are commonly administered before death. To assess their impact on the accuracy of predictions for the in-hospital mortality task, we analyzed the model outputs for those using medications as input (all modalities and medications only). We compared the performance across two subgroups: patients who received opiods and/or sedatives (group1) vs. those who did not (group2). Comparing AUROC values, we observed a slight increase in performance for group1 (0.942 vs. 0.930 in the all modality setting and 0.768 vs. 0.751 for medications only). However, a DeLong test found these differences not statistically significant (p=0.546, p=0.580). We believe this is due to the design of the dataset's benchmark task of in-hospital mortality prediction, which only includes events within the first 48 hours of hospitalization. Since death often occurs later, patients with shorter stays (including those who died within

this timeframe) are excluded. This leaves only a small subset for whom these medications might influence predictions, the ones who die immediately after the 48h timeframe.
⇒ We have incorporated this discussion on potential confounders in the medications.

Q8 Conducting more detailed ablation experiments could clarify the incremental benefit provided by each individual modality and help justify their inclusion more thoroughly.

A8 ⇒ We have now included comprehensive experimental results for all modality combinations on the MIMIC-IV dataset for predicting in-hospital mortality in Supplementary Table 5. Our systematic ablation study reveals distinct patterns in modality contributions. Clinical measurements (C) and X-ray images (X) provide the largest marginal improvements, with C|X achieving 0.875 AUROC compared to individual modalities. Medications (M) and ECG (E) show smaller individual contributions, with E yielding only 0.704 AUROC. However, the incremental benefits are additive: three-modality combinations (C|X|E: 0.851, C|X|M: 0.849) demonstrate consistent improvements over two-modality pairs except for C|X. The complete four-modality model (C|M|X|E) achieves 0.922 AUROC, representing a substantial improvement over the best three-modality combination, justifying the inclusion of all modalities despite their varying individual contributions.

Q9 Reporting detailed computational resource requirements (FLOPs, training/inference time, GPU usage, environmental impact) would be valuable for practical adoption and reproducibility.

A9 ⇒ We provide comprehensive computational resource analysis across all experimental configurations. AAll experiments were conducted on a single NVIDIA RTX 3090 GPU with 24 GB VRAM, where the Swin transformer with resolution 384×384 demands 464.692 GFLOPs per forward pass. Inference times demonstrate real-time applicability, it achieves 0.074 seconds for all modalities. Environmental impact was quantified using Carbon Tracker [1]. For the CDSL dataset, single modality training required approximately 6 minutes 30 seconds, consuming 0.076 kWh energy and generating 28.82g CO_2 emissions. Complete multimodal training extended to 10 minutes 30 seconds with 0.129 kWh energy consumption and 49.05g CO_2 emissions. On the larger MIMIC dataset, single modality training took 9 minutes 45 seconds, consuming 0.128 kWh and producing 48.77g CO_2. Full multimodal training required approximately 27 minutes with 0.224 kWh energy consumption and 85.46g CO_2 emissions.

Q10 Minor improvements in explicitly linking model insights (such as highlighted attention regions) to clinically meaningful indicators or standard clinical assessments would strengthen the clinical relevance and interpretability.

A10 Thank you for this valuable idea to strengthen clinical relevance and interpretability.
⇒ After consulting an expert physician, we have added clinical interpretations linking the model's attention patterns to established clinical indicators. Specifically, we explain how the model's focus on vital signs (respiratory rate, blood pressure, heart rate) aligns with core hemodynamic parameters that are critical mortality predictors in ICU patients. We describe the attention on vasopressors and inotropes as clinically meaningful since these medications indicate severe hemodynamic instability associated with increased mortality risk. Additionally, we interpret the model's attention to implanted pacemakers as recognition of significant cardiovascular comorbidity factors. We also identify the diffuse ECG attention pattern as a potential area for future model improvements through enhanced cardiac-specific feature engineering.

Table 1 Frequency distribution analysis across all clinical variables in the dataset. Statistics show sampling frequencies in samples per hour.

Variable	Max	Mean	Median	P95	P99	StdDev	N_intervals
O2 saturation pulseoxymetry	60.000	2.788	1.000	8.571	60.000	7.497	3939
Heart Rate	60.000	2.889	1.000	12.000	60.000	7.742	4059
Respiratory Rate	60.000	2.768	1.000	10.000	60.000	7.316	3998
GCS - Eye Opening	60.000	0.475	0.250	1.000	1.146	2.086	836
GCS - Verbal Response	60.000	0.472	0.250	1.000	1.095	2.080	839
GCS - Motor Response	60.000	0.477	0.250	1.000	1.202	2.090	833
Non Invasive Blood Pressure systolic	60.000	1.802	1.000	4.000	15.000	3.636	3065
Non Invasive Blood Pressure diastolic	60.000	1.802	1.000	4.000	15.000	3.636	3065
Non Invasive Blood Pressure mean	60.000	1.803	1.000	4.000	15.000	3.645	3061
Inspired O2 Fraction	30.000	0.744	0.302	2.000	9.029	2.295	216
PAR-Oxygen saturation	30.000	11.678	4.000	27.400	29.480	13.012	3
Pulmonary Artery Pressure systolic	20.000	2.246	1.000	4.257	14.340	2.547	223
Pulmonary Artery Pressure diastolic	20.000	2.246	1.000	4.257	14.340	2.547	223
Pulmonary Artery Pressure mean	20.000	2.245	1.000	4.393	14.490	2.574	218
Hemoglobin	15.000	0.399	0.121	1.014	6.510	1.381	167
Glucose (whole blood)	15.000	0.931	0.789	2.091	3.464	1.549	95
Temperature Fahrenheit	6.000	0.396	0.250	1.000	3.905	0.526	839
Lactic Acid	2.727	0.490	0.297	1.500	2.500	0.522	101
Arterial O2 pressure	2.727	0.610	0.448	1.500	2.500	0.540	107
Arterial CO2 Pressure	2.727	0.610	0.448	1.500	2.500	0.540	107
PH (Arterial)	2.727	0.604	0.444	1.500	2.500	0.537	109
WBC	1.395	0.185	0.111	0.602	1.143	0.222	153
INR	1.395	0.215	0.123	0.823	1.313	0.265	111
Potassium (serum)	0.938	0.150	0.116	0.360	0.690	0.130	195
Sodium (serum)	0.938	0.151	0.117	0.364	0.692	0.131	193
Creatinine (serum)	0.938	0.143	0.115	0.312	0.671	0.119	189
BUN	0.938	0.145	0.114	0.315	0.671	0.124	189
PH (Venous)	0.571	0.178	0.158	0.288	0.515	0.117	21
Total Bilirubin	0.286	0.119	0.116	0.251	0.277	0.072	40
ALT	0.286	0.121	0.116	0.253	0.278	0.073	37
Troponin-T	0.226	0.136	0.145	0.226	0.226	0.044	19
CK (CPK)	0.200	0.113	0.102	0.189	0.198	0.050	12
Direct Bilirubin	0.199	0.186	0.186	0.197	0.198	0.011	3

Max = Maximum frequency, Mean = Average frequency, Median = Median frequency, P95 = 95th percentile, P99 = 99th percentile, StdDev = Standard deviation, N_intervals = Number of measurement intervals analyzed. All frequency values are in samples per hour.

References

[1] Anthony LFW, Kanding B, Selvan R (2020) Carbontracker: Tracking and predicting the carbon footprint of training deep learning models. ICML Workshop on Challenges in Deploying and monitoring Machine Learning Systems, arXiv:2007.03051

[2] Harutyunyan H, Khachatrian H, Kale DC, Ver Steeg G, Galstyan A (2019) Multitask learning and benchmarking with clinical time series data. Scientific Data 6(1):96. https://doi.org/10.1038/s41597-019-0103-9

[3] Hayat N, Geras KJ, Shamout FE (2022) MedFuse: Multi-modal Fusion with Clinical Time-Series Data and Chest X-Ray Images. In: Proceedings of the 7th Machine Learning for Healthcare Conference, pp 479–503, URL https://proceedings.mlr.press/v182/hayat22a.html

[4] Irvin J, Rajpurkar P, Ko M, Yu Y, Ciurea-Ilcus S, Chute C, Marklund H, Haghgoo B, Ball R, Shpanskaya K, et al (2019) Chexpert: A large chest radiograph dataset with uncertainty labels and expert comparison. In: AAAI conference on artificial intelligence, pp 590–597

[5] Khader F, Kather JN, Müller-Franzes G, Wang T, Han T, Arasteh ST, Hamesch K, Bressem K, Haarburger C, Stegmaier J, Kuhl C, Nebelung S, Truhn D (2023) Medical transformer for multimodal survival prediction in intensive care: integration of imaging and non-imaging data. Nature Scientific Reports 13(1):10666. https://doi.org/10.1038/s41598-023-37835-1

[6] Li Z, Li S, Yan X (2023) Time Series as Images: Vision Transformer for Irregularly Sampled Time Series. In: Proceedings of the 37th Conference on Neural Information Processing Systems (NeurIPS), URL https://openreview.net/forum?id=ZmeAoWQqe0

[7] Liu Y, Ott M, Goyal N, Du J, Joshi M, Chen D, Levy O, Lewis M, Zettlemoyer L, Stoyanov V (2019) RoBERTa: A Robustly Optimized BERT Pretraining Approach. https://doi.org/10.48550/arXiv.1907.11692

[8] Liu Z, Lin Y, Cao Y, Hu H, Wei Y, Zhang Z, Lin S, Guo B (2021) Swin Transformer: Hierarchical Vision Transformer using Shifted Windows. In: International Conference on Computer Vision, https://doi.org/1048550/arXiv.2103.14030

[9] Ritoré Á, Oprescu AM, Estirado Bronchalo A, Armengol de la Hoz MÁ (2024) COVID data for shared learning (CDSL): A comprehensive, multimodal COVID-19 dataset from HM hospitales. https://doi.org/10.13026/1176-6c44

[10] Wang M, Fan S, Li Y, Xie Z, Chen H (2025) Missing-modality enabled multi-modal fusion architecture for medical data. Journal of Biomedical Informatics 164:104796. https://doi.org/10.1016/j.jbi.2025.104796
