# Supplementary material for: Visual prompt engineering for multimodal and irregularly sampled medical data
Source: Commun Med (Lond). 2026 Sep 1;6:474. doi: 10.1038/s43856-026-01817-x (PMC13534428; doi:10.1038/s43856-026-01817-x)
Supplement: Supplementary file 2 [file 43856_2026_1817_MOESM2_ESM.pdf]

Supplementary Information

Hyperparameter

Supplementary Table 1: Hyperparameter of ViTiMM. For training more than one modality the batch size had to be reduced to 4. Training was performed on two Nvidia RTX 3090 Ti. 3 epochs were sufficient for model training, afterwards slight overfitting could be observed similar to Li et al. [35].

**Experiment**	**Model**	**Image Size**	**Epochs**	**Optimizer**	**LRs**	**L_2-Reg.**	**Batch Size**
In-hospital Mortality	Swin-Large	384	3	AdamW	$10^{-5}, 5 \times 10^{-6}, 10^{-6}$	$3e-8$	8 (4)
Phenotyping	Swin-Large	384	3	AdamW	10^{-5}	$3e-8$	8 (4)
Decompensation	Swin-Large	384	3	AdamW	10^{-5}	$3e-8$	8 (4)

MIMIC Input Parameter: Clinical Measurements and Medications

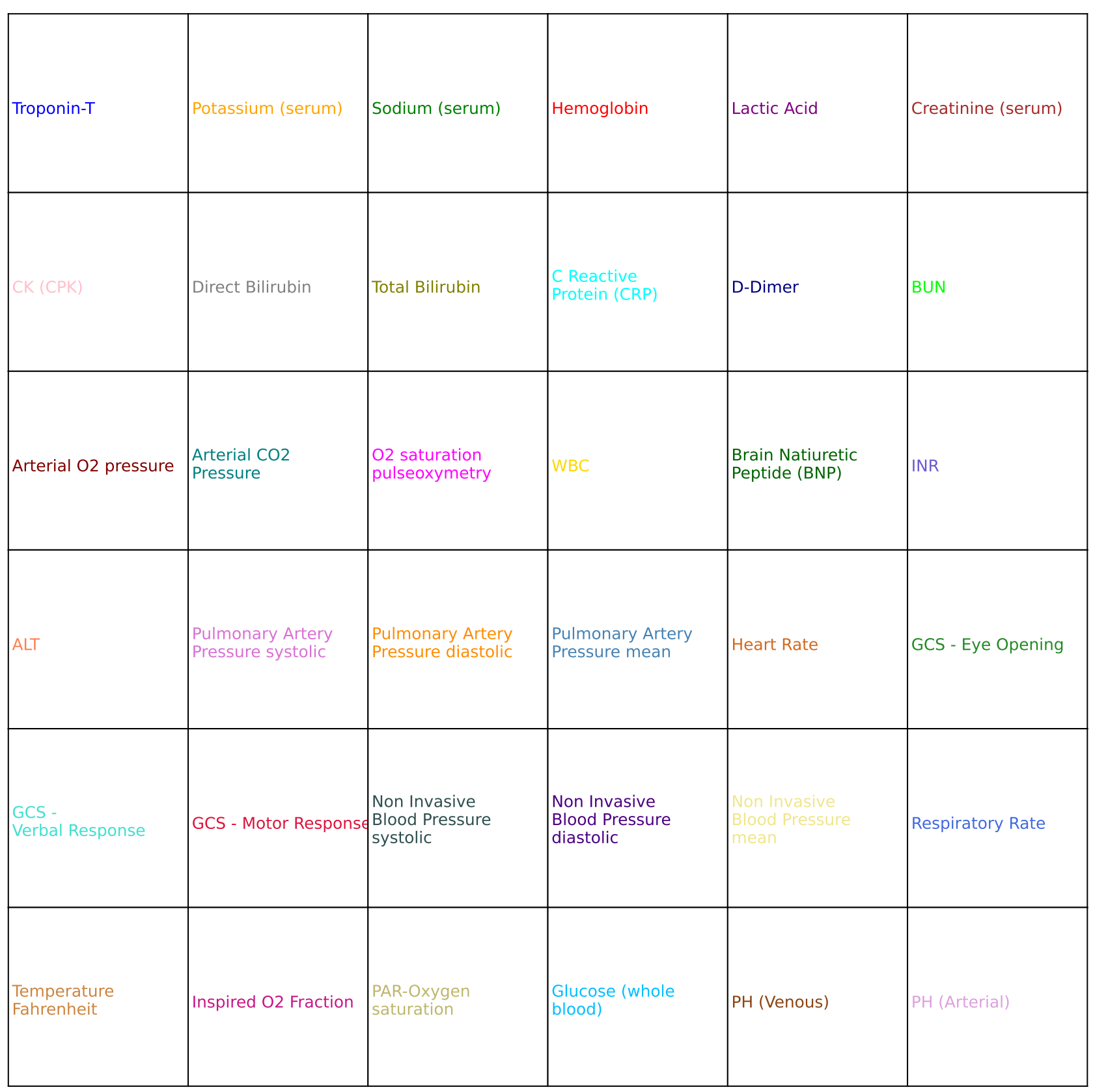

Supplementary Figure 1: All clinical measurements (C) in their respective field color in the line plots.

Antiarrhythmics<br>Amiodarone 600/500<br>Amiodarone<br>Lidocaine<br>Adenosine<br>Procainamide	Electrolyte Supplements<br>Potassium Chloride<br>Calcium Gluconate<br>Calcium Gluconate (CRRT)<br>K Phos<br>Na Phos	Beta-Blockers<br>Metoprolol<br>Labetalol<br>Esmolol	Diuretics<br>Furosemide (Lasix)<br>Furosemide (Lasix) 250/50<br>Mannitol
Cardiac Glycosides<br>Digoxin (Lanoxin)	Insulins<br>Insulin - Regular<br>Insulin - Humalog<br>Insulin - Glargine<br>Insulin - NPH<br>Insulin - 70/30<br>Insulin - Novolog<br>Insulin - Humalog 75/25	Opioid Analgesics<br>Fentanyl<br>Fentanyl (Concentrate)<br>Hydromorphone (Dilaudid)<br>Morphine Sulfate<br>Meperidine (Demerol)<br>Methadone Hydrochloride	Sedatives and Anesthetics<br>Propofol<br>Midazolam (Versed)<br>Dexmedetomidine (Precedex)<br>Lorazepam (Ativan)<br>Ketamine<br>Diazepam (Valium)<br>Pentobarbital
Thrombolytics<br>Alteplase (TPA)	Vasopressors and Inotropes<br>Norepinephrine<br>Phenylephrine<br>Epinephrine<br>Dopamine<br>Vasopressin<br>Milrinone<br>Dobutamine<br>Isuprel		

Supplementary Figure 2: All medications (M) in their respective field color in the line plots.

Supplementary Table 2: MIMIC Summary of Clinical Parameters with Statistics, Unit, and Missing Data. CK (CPK): Creatine Kinase, CRP: C Reactive Protein, WBC: White Blood Cells, BNP: Brain Natriuretic Peptide, INR: International Normalized Ratio, ALT: Alanine Aminotransferase, SYS: systolic, DIA: diastolic, PAP: Pulmonary Artery Pressure, NIBP: Non Invasive Blood Pressure, PAR: partial.

**Variable**	**Mean**	**Std**	**Lower**	**Upper**	**Unit**	**Missing (%)**
Troponin-T	0.70	1.34	0.00	0.01	ng/mL	47.18
Potassium (serum)	4.08	0.54	3.30	5.10	mmol/L	0.01
Sodium (serum)	139.09	5.05	133.00	145.00	mmol/L	0.01
Hemoglobin	9.90	1.93	12.00	16.00	g/dL (M)	0.01
Lactic Acid	2.52	2.04	0.50	2.00	mmol/L	14.89
Creatinine (serum)	1.38	1.22	0.40	1.10	mg/dL (M)	0.01
CK (CPK)	1768.03	4669.05	NaN	NaN	U/L	50.25
Direct Bilirubin	3.55	3.93	0.00	0.30	mg/dL	86.33
Total Bilirubin	2.12	3.38	0.00	1.50	mg/dL	29.20
CRP	73.18	77.64	0.00	5.00	mg/L	94.48
D-Dimer	8042.00	6100.88	0.00	0.50	µg/mL	97.69
BUN	28.31	22.58	7.00	20.00	mg/dL	0.01
Arterial O2 pressure	143.81	81.18	85.00	105.00	mmHg	20.95
Arterial CO2 Pressure	40.85	8.94	35.00	45.00	mmHg	20.95
O2 pulseoxymetry	96.19	2.24	95.00	100.00	%	0.00
WBC	11.71	5.59	0.00	5.00	cells/µL	0.01
BNP	6063.74	9257.13	0.00	100.00	pg/mL	89.44
INR	1.46	0.54	0.90	1.10	-	3.46
ALT	203.73	669.92	0.00	40.00	U/L	28.99
PAP-SYS	35.89	8.94	15.00	30.00	mmHg	86.44
PAP-DIA	18.44	5.37	8.00	15.00	mmHg	86.44
PAP-MEAN	25.13	6.30	10.00	20.00	mmHg	86.23
Heart Rate	85.75	17.27	60.00	100.00	beats/min	0.00
GCS-Eye Opening	-	-	1.00	4.00	-	0.00
GCS-Verbal Response	-	-	1.00	5.00	-	0.00
GCS-Motor Response	-	-	1.00	6.00	-	0.00
NIBP-SYS	119.52	20.51	90.00	120.00	mmHg	0.34
NIBP-DIA	65.51	14.06	60.00	80.00	mmHg	0.34
NIBP-MEAN	78.85	14.09	70.00	100.00	mmHg	0.34
Respiratory Rate	19.80	5.14	12.00	20.00	breaths/min	0.00
Temperature	98.51	1.02	97.00	99.50	°F	0.49
Inspired O2 Fraction	44.90	10.28	0.21	1.00	-	17.73
PAR-O2 saturation	-	-	95.00	100.00	%	82.69
Glucose (whole blood)	139.71	42.06	70.00	105.00	mg/dL	49.85
PH (Venous)	7.36	0.09	7.31	7.41	-	47.69
PH (Arterial)	7.38	0.08	7.35	7.45	-	20.55

Supplementary Table 3: MIMIC Medication Summary with Categories, Statistics, and Missing Values

Category	Medication	Mean	Std	Unit	Missing (%)
Antiarrhythmics	Amiodarone 600/500	320.05	164.60	mg	95.72
	Amiodarone	165.50	76.72	mg	96.47
	Lidocaine	541.49	574.92	mg	99.63
	Adenosine	6.93	2.61	mg	99.62
	Procainamide	656.89	312.71	mg	99.97
Electrolyte Supplements	Potassium Chloride	18.25	20.57	mEq	59.56
	Calcium Gluconate	2.04	1.40	g	63.90
	Calcium Gluconate (CRRT)	17.01	50.70	g	97.98
	K Phos	19.23	10.72	mmol	94.47
	Na Phos	19.82	10.71	mmol	95.28
Beta-Blockers	Metoprolol	5.14	2.01	mg	85.42
	Labetalol	46.41	116.89	mg	93.60
	Esmolol	848.74	769.93	mg	98.63
Diuretics	Furosemide (Lasix)	42.81	39.13	mg	67.87
	Furosemide (Lasix) 250/50	114.20	103.18	mg	95.47
	Mannitol	46.39	26.35	g	98.81
Cardiac Glycosides	Digoxin (Lanoxin)	14.69	49.34	mg	99.16
Insulins	Insulin - Regular	7.86	16.23	units	69.96
	Insulin - Humalog	4.57	4.37	units	81.54
	Insulin - Glargine	21.14	15.04	units	86.51
	Insulin - NPH	18.15	13.08	units	98.98
	Insulin - 70/30	22.26	12.90	units	99.71
	Insulin - Novolog	4.95	3.15	units	99.74
	Insulin - Humalog 75/25	14.82	6.68	units	99.94
Opioid Analgesics	Fentanyl	45.02	24.31	mcg	62.03
	Fentanyl (Concentrate)	0.59	0.85	mg	68.36
	Hydromorphone (Dilaudid)	0.82	2.02	mg	83.97
	Morphine Sulfate	2.89	5.77	mg	83.85
	Meperidine (Demerol)	13.84	3.88	mg	98.75
	Methadone Hydrochloride	13.75	8.71	mg	99.85
Sedatives and Anesthetics	Propofol	294.35	341.89	mg	54.55
	Midazolam (Versed)	8.60	18.76	mg	78.26
	Dexmedetomidine (Precedex)	90.21	101.66	mcg	91.29
	Lorazepam (Ativan)	1.18	1.14	mg	87.72
	Ketamine	58.58	38.57	mg	99.76
	Diazepam (Valium)	7.20	4.57	mg	99.72
	Pentobarbital	508.17	749.81	mg	99.94
Thrombolytics	Alteplase (TPA)	20.68	24.19	mg	99.78
Vasopressors and Inotropes	Norepinephrine	1.21	1.98	mg	74.43
	Phenylephrine	7.93	15.50	mg	74.93
	Epinephrine	0.87	1.81	mg	95.28
	Dopamine	83.34	117.36	mg	96.44
	Vasopressin	18.72	20.28	units	93.20
	Milrinone	11.06	7.48	mg	97.82
	Dobutamine	110.95	109.13	mg	98.37
	Isuprel	0.39	0.47	mg	99.91

CDSL Input Parameter: Clinical Measurements and Medications

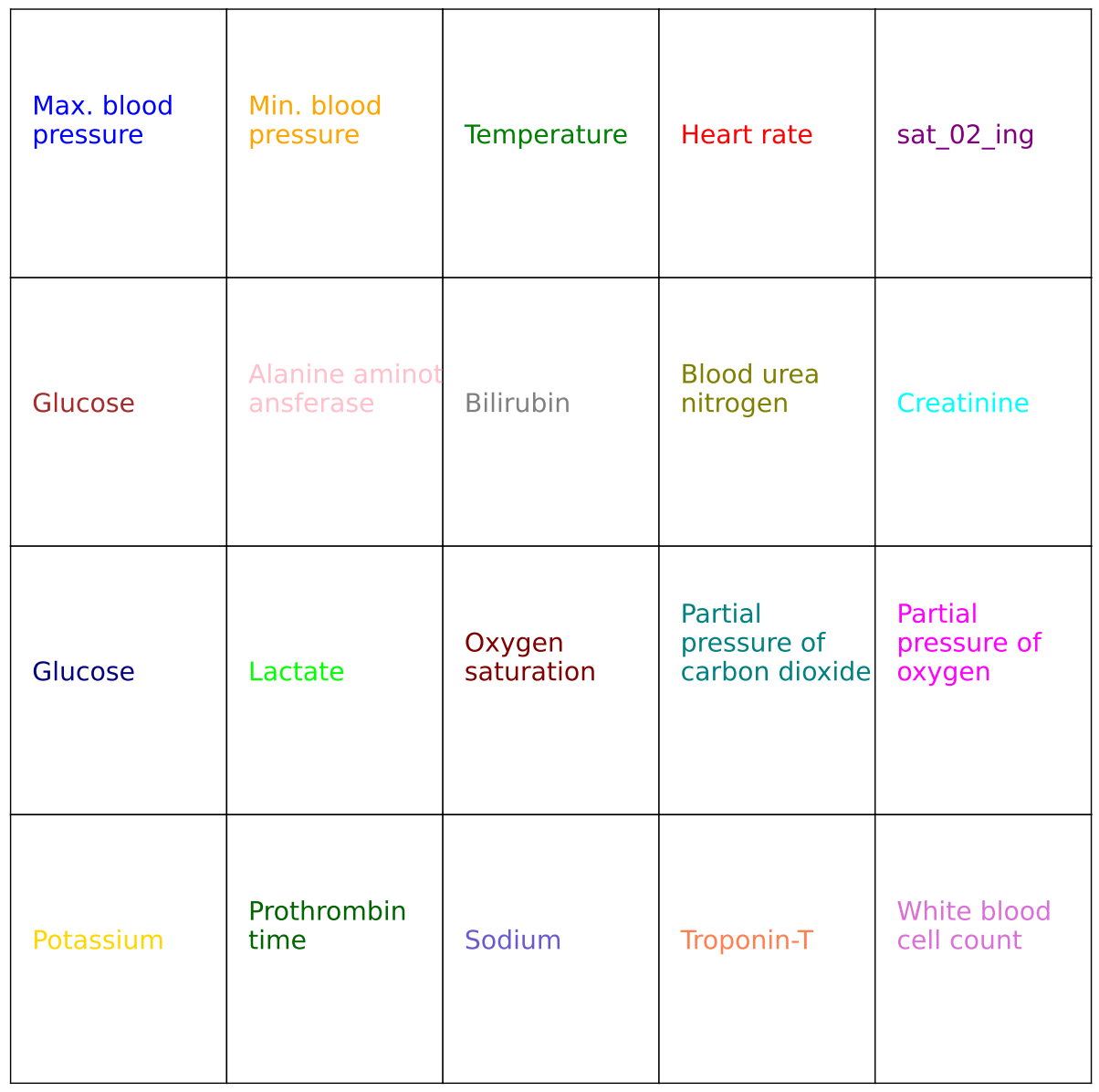

Supplementary Figure 3: Clinical measurement categories for CDSL dataset.

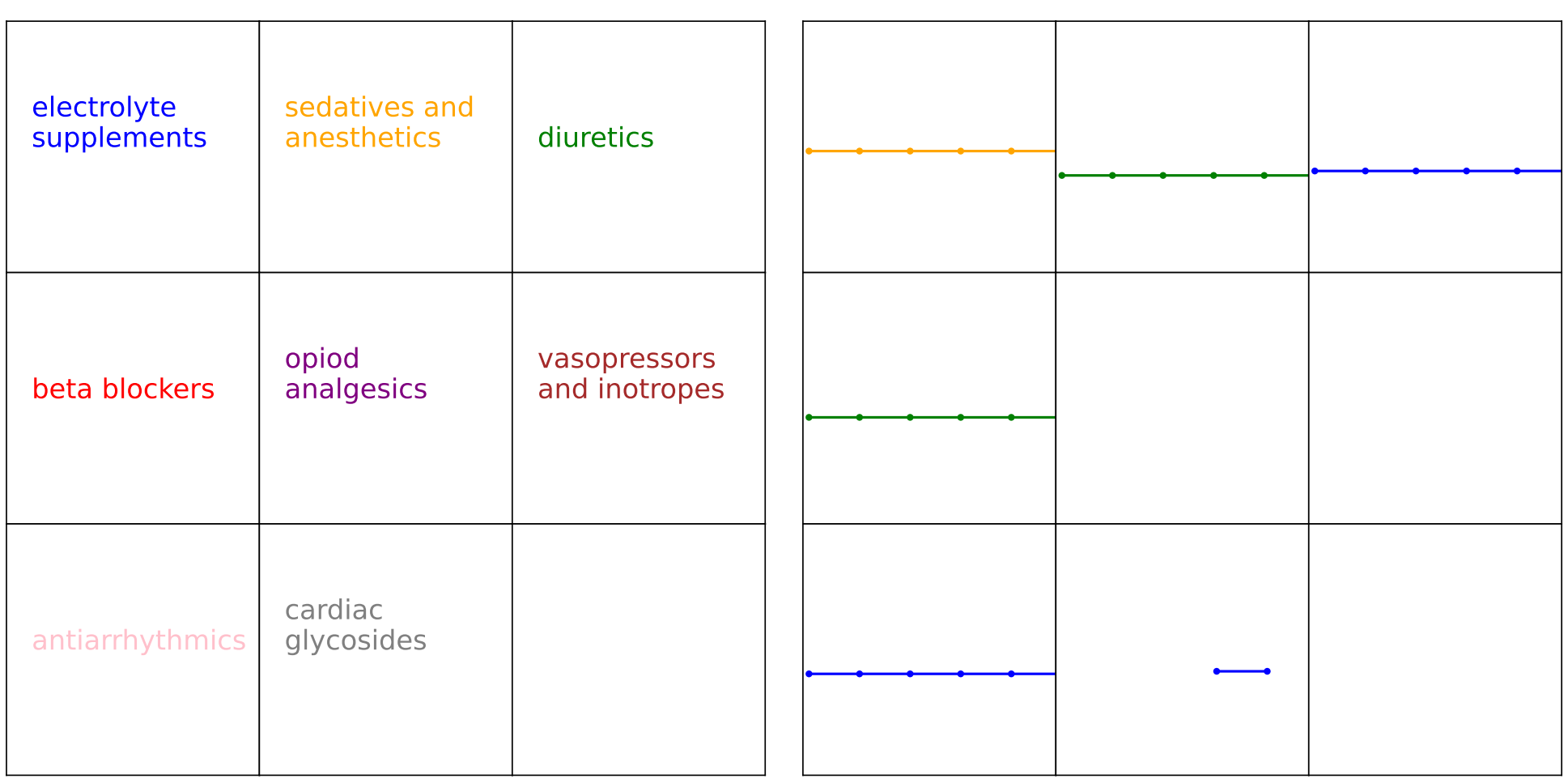

(a) Medications (b) Example Plot

Supplementary Figure 4: Medication caterogies and example plot for CDSL dataset.

Supplementary Table 4: CDSL Summary of Clinical Parameters with Statistics, Unit, and Missing Data.

**Variable**	**Mean**	**Std**	**Lower**	**Upper**	**Unit**	**Missing (%)**
BP max admission	124.32	19.44	90	140	mmHg	0.0
BP min admission	71.38	12.08	60	90	mmHg	0.0
Temperature admission	36.26	0.72	36.1	37.2	°C	0.0
Heart rate admission	75.71	14.60	60	100	bpm	0.0
O_2 saturation admission	95.03	33.35	95	100	%	0.0
Glucose admission	—	—	70	100	mg/dL	0.0
Alanine aminotransferase	52.26	80.66	7	56	U/L	7.4
Bilirubin	0.69	0.90	0.1	1.2	mg/dL	28.5
Blood urea nitrogen	3566.00	8786.12	7	20	mg/dL	96.2
Creatinine	0.97	0.59	0.6	1.3	mg/dL	5.4
Glucose	125.81	52.09	70	100	mg/dL	6.7
Lactate	1.94	0.88	0.5	2.2	mmol/L	75.7
Oxygen saturation	94.20	7.80	95	100	%	80.7
Partial pressure CO2	44.83	10.14	35	45	mmHg	80.4
Partial pressure O2	78.87	43.09	75	100	mmHg	65.7
Potassium	4.32	0.60	3.5	5.1	mEq/L	0.073
Prothrombin time	13.81	6.82	11	13	seconds	22.5
Sodium	138.64	5.35	136	145	mEq/L	6.8
Troponin-T	45.73	228.38	0	14	ng/L	76.8
White blood cell count	8.30	5.25	4.5	11.0	$\times 10^3/\mu L$	4.0
pH	7.40	0.08	7.35	7.45	—	93.5

Supplementary Table 5: CDSL Medication Summary with Categories, Statistics, and Missing Values Part 1. All units are normalized to mg.

**Category**	**Drug Name**	**Mean**	**Std**	**Unit**	**Missing (%)**
Antiarrhythmics	Amiodarona	385.90	320.56	mg	99.01
	Apocard	171.43	65.87	mg	99.21
	Biocoryl	5000.00	—	mg	99.97
	Cordarone	450.00	—	mg	99.97
	Fenitoina	750.00	—	mg	99.97
	Lidocaina	146.67	83.38	mg	99.57
	Rytmonorm	407.14	207.02	mg	99.80
	Trangorex	481.06	392.31	mg	98.36
Beta Blockers	Atenolol	100.00	—	mg	99.97
	Beloken	122.00	80.12	mg	99.86
	Bisoprolol	6.25	2.73	mg	90.50
	Carvedilol	21.02	16.99	mg	97.96
	Lobivon	10.00	—	mg	99.97
	Sotapor	160.00	80.00	mg	99.92
	Sumial	26.79	21.09	mg	99.21
	Tenormin	69.84	40.04	mg	96.43
	Trandate	370.59	413.47	mg	99.55
Cardiac Glycosides	Digoxina	0.41	0.31	mg	98.30
Diuretics	Aldactone	43.81	32.67	mg	97.42
	Ameride	50.00	0.00	mg	99.43
	Furosemida	83.22	153.85	mg	86.56
	Hidrosaluretil	53.73	14.54	mg	96.20
	Higrotona	50.00	0.00	mg	99.60
	Inspra	29.55	10.11	mg	99.69
	Samsca	15.00	—	mg	99.97
	Seguril	46.89	17.41	mg	91.44
	Suero	50000.00	—	mg	93.20
	Sutril	6.85	3.22	mg	98.70
	Tertensif	2.50	0.00	mg	99.80
Electrolyte Supplements	Caosina	3152.17	1721.75	mg	99.35
	Cloruro	533.59	1732.18	mg	17.86
	Fosfato	7120.00	0.00	mg	98.24
	Magnogene	106.83	67.10	mg	98.19
	Mastical	2083.33	1355.82	mg	99.49
	Osvical	675.00	212.13	mg	99.77
	Potasion	1294.74	706.20	mg	98.38
	Sulfato	3000.00	0.00	mg	99.94
	Suplecal	1636.30	860.08	mg	98.47
Opioid Analgesics	Dolantina	150.00	142.46	mg	99.49
	Durogesic	0.04	0.02	mg	99.04
	Effentora	0.27	0.29	mg	99.92
	Fentanest	0.80	0.69	mg	97.42
	Morfina	36.94	31.88	mg	90.02
	MST	33.33	20.66	mg	99.86
	Palexia	83.33	43.78	mg	99.83
	Sevredol	18.33	11.69	mg	99.83
	Tramadol	176.96	90.84	mg	97.39

Supplementary Table 6: CDSL Medication Summary with Categories, Statistics, and Missing Values Part 2. All units are normalized to mg.

Category	Drug Name	Mean	Std	Unit	Missing (%)
Sedatives and Anesthetics	Alprazolam	1.02	1.17	mg	98.84
	Atarax	25.00	0.00	mg	99.69
	Bromazepam	2.20	1.03	mg	94.30
	Circadin	2.00	0.00	mg	99.94
	Dexdor	4.00	1.41	mg	99.94
	Dexmedetomidina	1.31	0.64	mg	98.02
	Diazepan	9.35	24.58	mg	97.82
	Diprivan	4200.00	1643.17	mg	99.89
	Distraneurine	226.91	77.67	mg	99.69
	Dormodor	90.00	—	mg	99.97
	Hypnomidate	25.00	11.00	mg	99.43
	Ketolar	2000.00	1500.00	mg	99.92
	Lorazepam	1.11	0.43	mg	72.14
	Lormetazepam	1.21	0.46	mg	96.15
	Midazolam	146.79	166.25	mg	93.45
	Propofol	3500.39	3181.10	mg	95.49
	Remifentanilo	8.26	3.07	mg	98.04
	Sedotime	22.50	10.61	mg	99.94
	Trankimazin	0.75	0.35	mg	99.94
	Tranxilium	28.43	26.08	mg	98.72
	Valium	29.67	31.24	mg	98.30
	Zolpidem	10.18	1.34	mg	98.41
Vasopressors and Inotropes	Adrenalina	8.67	7.64	mg	99.66
	Aleudrina	1.20	0.72	mg	99.92
	Dobutamina	650.00	285.04	mg	99.86
	Dopamina	428.57	75.59	mg	99.80
	Gutron	25.00	—	mg	99.97
	Noradrenalina	45.24	36.00	mg	99.40
	Norages	53.66	31.77	mg	96.32
	Simdax	12.50	—	mg	99.97

Results Phenotyping per Class

Supplementary Table 7: AUROC values for all methods and modality combinations for task *phenotyping* per class. Additionally, the prevalence of each class in train- and testset is reported. All = $C|M|X|E$.

Phenotype	Prevalence		MeTra			MedFuse			ViTiMM (ours)					
	Train	Test	C	X	$C\|X$	C	X	$C\|X$	C	X	M	E	$C\|X$	All
Acute and unspecified renal failure	0.371	0.358	0.707	0.679	0.713	0.741	0.660	0.753	0.870	0.743	0.746	0.729	0.869	0.872
Acute cerebrovascular disease	0.099	0.095	0.897	0.683	0.889	0.881	0.644	0.888	0.870	0.758	0.768	0.662	0.860	0.878
Acute myocardial infarction	0.090	0.098	0.710	0.650	0.700	0.724	0.644	0.717	0.891	0.805	0.773	0.704	0.912	0.903
Cardiac dysrhythmias	0.413	0.405	0.603	0.666	0.643	0.618	0.654	0.681	0.703	0.698	0.705	0.676	0.709	0.705
Chronic kidney disease	0.250	0.243	0.711	0.707	0.739	0.737	0.687	0.776	0.903	0.757	0.745	0.739	0.906	0.907
COPD and bronchiectasis	0.172	0.151	0.623	0.726	0.720	0.632	0.671	0.690	0.695	0.751	0.652	0.588	0.754	0.742
Complications of procedures	0.264	0.277	0.670	0.606	0.653	0.660	0.546	0.664	0.666	0.648	0.662	0.646	0.666	0.677
Conduction disorders	0.111	0.119	0.680	0.799	0.789	0.626	0.707	0.796	0.752	0.850	0.751	0.801	0.847	0.874
Congestive heart failure	0.336	0.307	0.710	0.743	0.759	0.731	0.726	0.798	0.816	0.813	0.807	0.815	0.828	0.819
Coronary atherosclerosis	0.317	0.326	0.727	0.734	0.745	0.725	0.681	0.743	0.807	0.788	0.779	0.726	0.831	0.809
Diabetes w/ complications	0.119	0.117	0.810	0.583	0.817	0.829	0.567	0.827	0.826	0.809	0.819	0.818	0.823	0.852
Diabetes w/o complication	0.207	0.221	0.673	0.598	0.692	0.699	0.547	0.717	0.728	0.706	0.720	0.681	0.714	0.719
Disorders of lipid metabolism	0.397	0.414	0.637	0.669	0.654	0.663	0.630	0.675	0.688	0.666	0.682	0.656	0.687	0.684
Essential hypertension	0.435	0.437	0.598	0.613	0.645	0.643	0.611	0.681	0.728	0.662	0.665	0.633	0.725	0.726
Fluid and electrolyte disorders	0.512	0.513	0.690	0.638	0.683	0.682	0.632	0.681	0.729	0.664	0.654	0.660	0.728	0.718
Gastrointestinal hemorrhage	0.072	0.071	0.667	0.594	0.663	0.697	0.597	0.690	0.741	0.664	0.660	0.579	0.727	0.777
Hypertension w/ complications	0.225	0.221	0.686	0.686	0.723	0.719	0.691	0.770	0.886	0.752	0.749	0.725	0.882	0.882
Other liver diseases	0.179	0.183	0.665	0.660	0.695	0.673	0.686	0.719	0.775	0.714	0.676	0.614	0.779	0.783
Other lower respiratory disease	0.140	0.144	0.532	0.604	0.546	0.566	0.551	0.580	0.555	0.582	0.528	0.462	0.576	0.573
Other upper respiratory disease	0.074	0.058	0.675	0.560	0.663	0.703	0.544	0.721	0.733	0.630	0.657	0.574	0.688	0.734
Pleurisy; pneumothorax; collapse	0.113	0.101	0.574	0.668	0.604	0.605	0.661	0.656	0.541	0.649	0.568	0.596	0.638	0.658
Pneumonia (except TB/STI)	0.228	0.232	0.696	0.677	0.727	0.721	0.633	0.739	0.722	0.707	0.661	0.656	0.732	0.742
Respiratory failure	0.350	0.360	0.792	0.731	0.801	0.785	0.675	0.788	0.804	0.757	0.746	0.724	0.805	0.809
Septicemia	0.265	0.264	0.760	0.685	0.764	0.762	0.675	0.766	0.817	0.789	0.773	0.781	0.821	0.821
Shock	0.224	0.217	0.785	0.707	0.782	0.809	0.676	0.807	0.889	0.870	0.873	0.874	0.892	0.891

Ablation Studies

Supplementary Table 8: Ablation Study for different modality combinations on the MIMIC-IV dataset for the task of in-hospital mortality prediction.
C: clinical measurements, X: CXR images, M: medications, E: electrocardiography.

**Modalities**	**ROCAUC**	**AUPRC**	**Bal.Acc.**
$C\|M$	0.817	0.465	0.714
$C\|E$	0.802	0.454	0.717
$X\|M$	0.823	0.504	0.741
$X\|E$	0.806	0.447	0.731
$M\|E$	0.731	0.351	0.665
$C\|X\|M$	0.849	0.530	0.766
$C\|X\|E$	0.851	0.545	0.770
$M\|X\|E$	0.819	0.496	0.747

Significance Tests

Supplementary Table 9: To assess the statistical significance for predicting in-hospital mortality on MIMIC, we conducted pairwise comparisons using the paired samples t-test [53].

		MeTra		MedFuse			MMFusion			ViTiMM					
		X	$C\|X$	C	X	$C\|X$	C	X	$C\|X$	C	X	M	E	$C\|X$	$C\|M\|X\|E$
MeTra	X	-	***	***	***	***	***	***	***	***	***	***	***	***	***
	$C\|X$		-	0.444	***	***	***	***	***	***	***	***	***	***	***
MedFuse	C			-	***	***	***	***	***	***	***	***	***	***	***
	X				-	0.253	***	***	***	***	***	***	***	***	***
	$C\|X$					-	***	***	***	***	***	***	***	***	***
MMFusion	C						-	***	***	***	***	***	***	***	***
	X							-	***	***	***	***	***	***	***
	$C\|X$								-	***	***	***	***	***	***
ViTiMM	C									-	0.104	***	***	***	***
	X										-	***	***	***	***
	M											-	0.143	***	***
	E												-	***	***
	$C\|X$													-	***

Supplementary Table 10: To assess the statistical significance for predicting phenotyping on MIMIC, we conducted pairwise comparisons using the paired samples t-test [53].

		MeTra		MedFuse			MMFusion			ViTiMM					
		X	$C\|X$	C	X	$C\|X$	C	X	$C\|X$	C	X	M	E	$C\|X$	$C\|M\|X\|E$
MeTra	X	-	***	***	0.717	0.362	***	***	***	0.005	***	***	0.004	***	***
	$C\|X$		-	***	***	***	***	***	***	***	***	***	***	***	0.279
MedFuse	C			-	***	***	***	***	***	***	***	***	***	***	***
	X				-	0.111	***	***	***	***	***	***	***	***	***
	$C\|X$					-	***	***	***	***	***	***	***	***	***
MMFusion	C						-	***	***	***	***	***	***	***	***
	X							-	***	***	***	***	***	***	***
	$C\|X$								-	***	***	***	***	***	***
ViTiMM	C									-	0.003	***	***	***	***
	X										-	0.283	***	***	***
	M											-	***	***	***
	E												-	0.001	0.062
	$C\|X$													-	***

Supplementary Table 11: To assess the statistical significance for predicting decompensation on MIMIC, we conducted pairwise comparisons using the paired samples t-test [53].

		MeTra		MedFuse			MMFusion			ViTiMM					
		X	$C\|X$	C	X	$C\|X$	C	X	$C\|X$	C	X	M	E	$C\|X$	$C\|M\|X\|E$
MeTra	X	-	***	***	***	***	***	***	***	***	***	***	***	***	***
	$C\|X$		-	***	***	***	***	***	***	***	***	***	***	***	***
MedFuse	C			-	***	***	***	***	***	***	***	***	***	***	***
	X				-	***	***	***	***	***	***	***	***	***	***
	$C\|X$					-	***	***	***	***	***	***	***	***	***
MMFusion	C						-	***	***	***	***	***	***	***	***
	X							-	***	***	***	***	***	***	***
	$C\|X$								-	***	***	***	***	***	***
ViTiMM	C									-	***	***	***	***	***
	X										-	***	***	***	***
	M											-	***	***	***
	E												-	***	***
	$C\|X$													-	***

Supplementary Table 12: To assess the statistical significance for predicting in-hospital mortality on CDSL, we conducted pairwise comparisons using the paired samples t-test [53].

		MeTra		MedFuse			MMFusion			ViTiMM				
		X	$C\|X$	C	X	$C\|X$	C	X	$C\|X$	C	X	M	$C\|X$	$C\|M\|X$
MeTra	X	-	***	***	***	***	***	***	***	***	***	***	***	***
	$C\|X$		-	***	***	***	***	***	***	***	***	***	***	***
MedFuse	C			-	***	***	***	***	***	***	***	***	***	***
	X				-	***	***	***	***	***	***	***	***	***
	$C\|X$					-	***	***	***	***	***	***	***	***
MMFusion	C						-	***	***	***	***	***	***	***
	X							-	***	***	***	***	***	***
	$C\|X$								-	***	***	***	***	***
ViTiMM	C									-	***	***	***	***
	X										-	***	***	***
	M											-	***	***
	$C\|X$												-	***

Attention Visualization

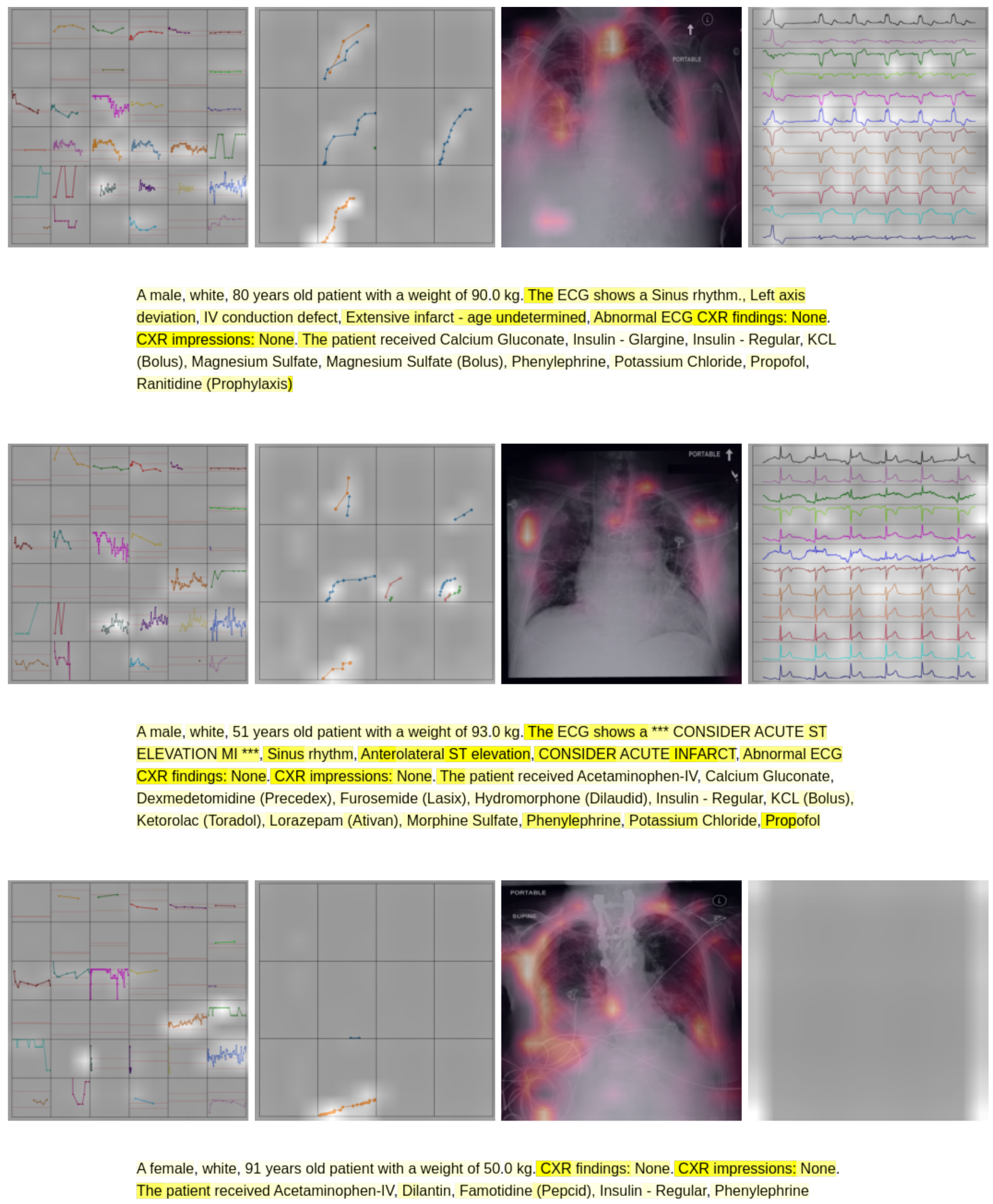

Supplementary Figure 5: Attention Maps of ViT for *In-hospital Mortality* prediction for `hadm_ids 30009123, 30090650, 30055897`. The patients survived the hospital stay, which the model correctly predicted in the top two cases but failed to do in the bottom one. However, with a predicted probability of 0.602, the model reflects a degree of uncertainty.

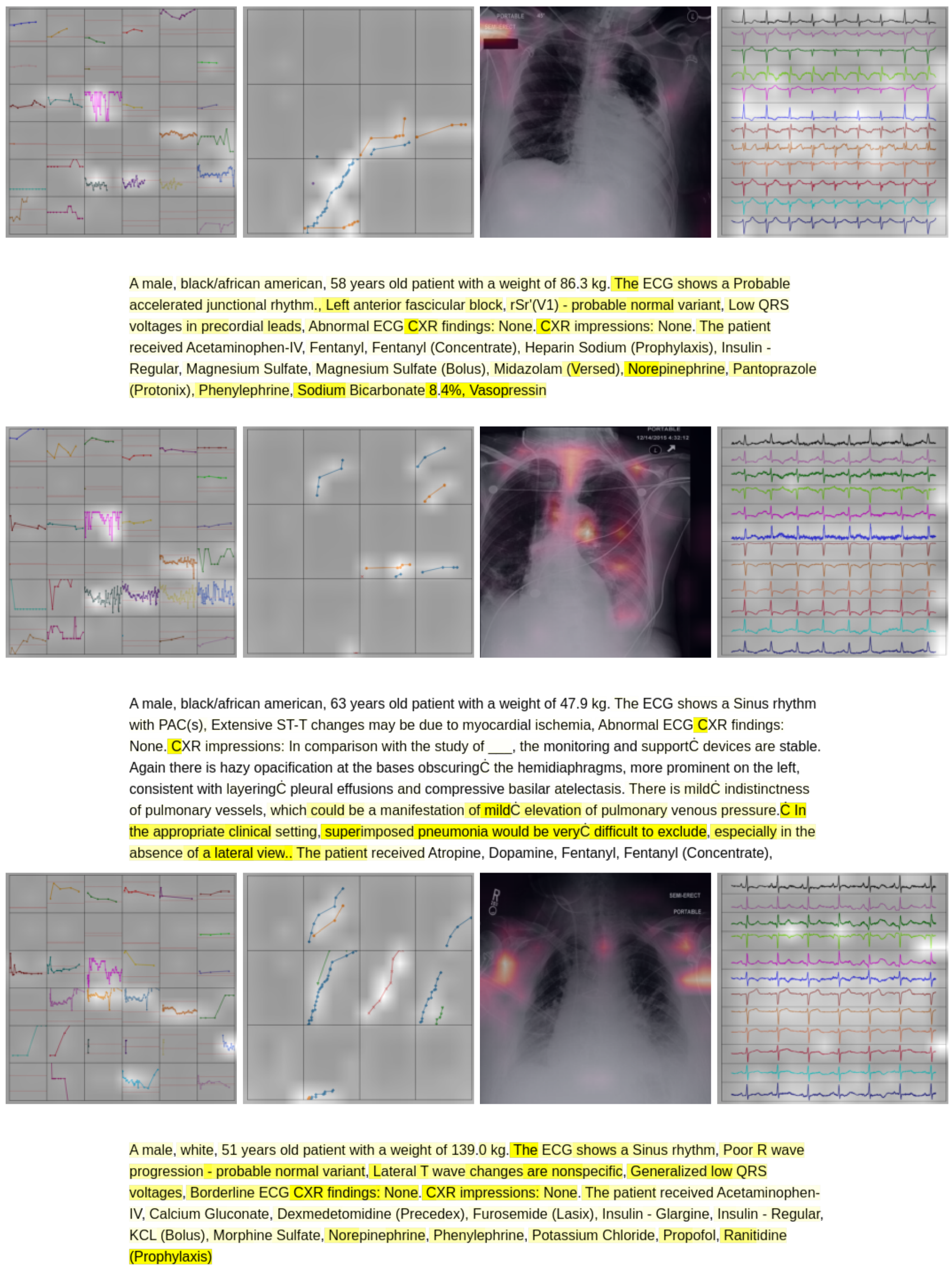

Supplementary Figure 6: Attention Maps of ViT for *In-hospital Mortality* prediction for `hadm_ids` `30204754, 30397772, 30596506`. The patients survived the hospital stay, which the model correctly predicted in the top two cases but again failed to do in the bottom one.

Information Loss Quantification

Supplementary Table 13: Frequency distribution analysis across all clinical variables in the dataset. Statistics show sampling frequencies in samples per hour.

**Variable**	**Max**	**Mean**	**Median**	**P95**	**P99**	**StdDev**	**N_intervals**
O2 saturation pulseoxymetry	60.000	2.788	1.000	8.571	60.000	7.497	3939
Heart Rate	60.000	2.889	1.000	12.000	60.000	7.742	4059
Respiratory Rate	60.000	2.768	1.000	10.000	60.000	7.316	3998
GCS - Eye Opening	60.000	0.475	0.250	1.000	1.146	2.086	836
GCS - Verbal Response	60.000	0.472	0.250	1.000	1.095	2.080	839
GCS - Motor Response	60.000	0.477	0.250	1.000	1.202	2.090	833
Non Invasive Blood Pressure systolic	60.000	1.802	1.000	4.000	15.000	3.636	3065
Non Invasive Blood Pressure diastolic	60.000	1.802	1.000	4.000	15.000	3.636	3065
Non Invasive Blood Pressure mean	60.000	1.803	1.000	4.000	15.000	3.645	3061
Inspired O2 Fraction	30.000	0.744	0.302	2.000	9.029	2.295	216
PAR-Oxygen saturation	30.000	11.678	4.000	27.400	29.480	13.012	3
Pulmonary Artery Pressure systolic	20.000	2.246	1.000	4.257	14.340	2.547	223
Pulmonary Artery Pressure diastolic	20.000	2.246	1.000	4.257	14.340	2.547	223
Pulmonary Artery Pressure mean	20.000	2.245	1.000	4.393	14.490	2.574	218
Hemoglobin	15.000	0.399	0.121	1.014	6.510	1.381	167
Glucose (whole blood)	15.000	0.931	0.789	2.091	3.464	1.549	95
Temperature Fahrenheit	6.000	0.396	0.250	1.000	3.905	0.526	839
Lactic Acid	2.727	0.490	0.297	1.500	2.500	0.522	101
Arterial O2 pressure	2.727	0.610	0.448	1.500	2.500	0.540	107
Arterial CO2 Pressure	2.727	0.610	0.448	1.500	2.500	0.540	107
PH (Arterial)	2.727	0.604	0.444	1.500	2.500	0.537	109
WBC	1.395	0.185	0.111	0.602	1.143	0.222	153
INR	1.395	0.215	0.123	0.823	1.313	0.265	111
Potassium (serum)	0.938	0.150	0.116	0.360	0.690	0.130	195
Sodium (serum)	0.938	0.151	0.117	0.364	0.692	0.131	193
Creatinine (serum)	0.938	0.143	0.115	0.312	0.671	0.119	189
BUN	0.938	0.145	0.114	0.315	0.671	0.124	189
PH (Venous)	0.571	0.178	0.158	0.288	0.515	0.117	21
Total Bilirubin	0.286	0.119	0.116	0.251	0.277	0.072	40
ALT	0.286	0.121	0.116	0.253	0.278	0.073	37
Troponin-T	0.226	0.136	0.145	0.226	0.226	0.044	19
CK (CPK)	0.200	0.113	0.102	0.189	0.198	0.050	12
Direct Bilirubin	0.199	0.186	0.186	0.197	0.198	0.011	3

Max = Maximum frequency, Mean = Average frequency, Median = Median frequency, P95 = 95th percentile, P99 = 99th percentile, StdDev = Standard deviation, N_intervals = Number of measurement intervals analyzed. All frequency values are in samples per hour.
